# Nonadditivity in public and inhouse data: implications for drug design

**DOI:** 10.1186/s13321-021-00525-z

**Published:** 2021-07-02

**Authors:** D. Gogishvili, E. Nittinger, C. Margreitter, C. Tyrchan

**Affiliations:** 1 Medicinal Chemistry, Research and Early Development, Respiratory and Immunology (R&I), BioPharmaceuticals R&D, AstraZeneca, Gothenburg, Sweden; 2 Computational Chemistry, Discovery Sciences, R&D, AstraZeneca, Gothenburg, Sweden; 3 Present Address: Department of Computer Science, Vrije Universiteit, De Boelelaan 1105, 1081 HV Amsterdam, The Netherlands

**Keywords:** Nonadditivity analysis, Structure-activity relationship, Matched molecular pair analysis, Experimental uncertainty, Machine learning, Support vector machine, Random forest

## Abstract

Numerous ligand-based drug discovery projects are based on structure-activity relationship (SAR) analysis, such as Free-Wilson (FW) or matched molecular pair (MMP) analysis. Intrinsically they assume linearity and additivity of substituent contributions. These techniques are challenged by nonadditivity (NA) in protein–ligand binding where the change of two functional groups in one molecule results in much higher or lower activity than expected from the respective single changes. Identifying nonlinear cases and possible underlying explanations is crucial for a drug design project since it might influence which lead to follow. By systematically analyzing all AstraZeneca (AZ) inhouse compound data and publicly available ChEMBL25 bioactivity data, we show significant NA events in almost every second assay among the inhouse and once in every third assay in public data sets. Furthermore, 9.4% of all compounds of the AZ database and 5.1% from public sources display significant additivity shifts indicating important SAR features or fundamental measurement errors. Using NA data in combination with machine learning showed that nonadditive data is challenging to predict and even the addition of nonadditive data into training did not result in an increase in predictivity. Overall, NA analysis should be applied on a regular basis in many areas of computational chemistry and can further improve rational drug design.

**Supplementary Information:**

The online version contains supplementary material available at 10.1186/s13321-021-00525-z.

## Introduction

The similarity and additivity principles represent the basis of various well-established areas in computer-aided drug design (CADD) such as Free-Wilson (FW) [1] analysis, two-dimensional (2D)/three-dimensional (3D) quantitative structure-activity relationship (QSAR) [2], matched molecular pair (MMP) [3] analysis, and computational scoring functions [4, 5]. Similarity and additivity are often implicitly assumed in CADD approaches in order to identify favorable molecular descriptors and predict the activity of new molecules. Otherwise chemists would have to synthesize and biologically evaluate every single molecule [6].

Yet, both these principles are subject to frequent disruptions. The exceptions to the similarity principle often complicate SAR analysis. So-called ‘activity cliffs’ refer to structurally very similar compound pairs with large alterations in potency [7–14]. Exceptions to linearity and additivity occur when the combination of substituents significantly boosts or decreases the biological activity of a ligand [15–19]. Nonadditivity (NA) may have several underlying reasons, including inconsistency in the binding pose of the central scaffold inside the pocket [20] and steric clashes [21]. Conformational changes in the binding pocket such as complete reorientation of the ligands alter the free energy of binding [15]. Furthermore, many nonadditive ‘magic methyl’ cases [13, 14, 22], i.e. attaching a simple alkyl fragment to a ligand that greatly increases the biological activity, can be explained by conformational changes as the so-called ‘ortho-effect’.

Additivity and NA of ligand binding have been studied for many years [23, 24] and can be perceived as a specific kind of interaction between functional groups [25, 26]. By analyzing public SAR data sets for strong NA (ΔΔpActivity > 2.0 log units) and respective X-ray structures, Kramer et al. showed that the cases of strong NA are underlined by changes in binding mode [15]. Babaoglu and Schoichet applied an inverse, deconstructive logic to structure-based drug design (SBDD) and by studying β-lactamase inhibitors demonstrated that fragments often do not recapitulate the binding affinity of the parent molecule [27]. The study of Miller and Wolfenden about substrate recognition demonstrated that the combination of distinct functional groups shows strong nonadditive behavior [28]. The work of Hajduk et al.[29] on stromelysin inhibitors and Congrive et al. [30] on CDK inhibitors showed that molecular affinity after combining a certain amount of functional groups is much higher than expected. Patel et al. examined various combinatorial libraries assayed on several different biological responses and concluded that only half of the data is additive [4]. McClure and colleagues developed a method to determine FW additivity in a combinatorial matrix of compounds (when multiple R groups are altered simultaneously; combinatorial analoging) and they intuitively explained the occurring NA by changes in binding mode without any structural validation [18, 19]. Water molecules are a major player in ligand−protein interactions by participating in extended hydrogen-bond networks [31]. Baum, Muley, and co-workers thoroughly analyzed the structural data and the reasons behind NA at the molecular level [17, 32] showing that NA can be the result of entropy and enthalpy profile changes, caused by hydrophobic interactions, hydrogen bonding and a loss of residual mobility of the bound ligands. In another study, Kuhn et al. proposed that internal hydrogen bonding gives rise to NA during compound optimization [33]. Gomez et al. explained NA caused by protein structural changes upon ligand binding [16]. According to these studies, instead of seeing NA as a problem, it should be interpreted as a hint towards key SAR features and variations in the binding modes. Identifying NA and understanding the reasons behind it is crucial for rational drug design since it provides valuable information about ligand-protein contacts and molecular recognition. NA analysis helps us to identify potential SAR outliers in a data set, ultimately suggesting interesting structural properties that might change the course of small molecule optimization. Importantly, NA might also be caused by experimental noise.

**Fig. 1:**
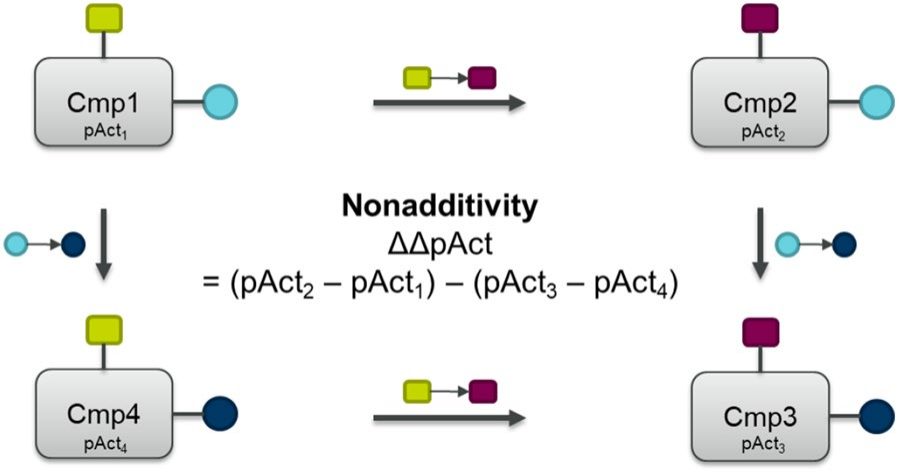
Schematic depiction of a double-transformation cycle consisting of four molecules. These four molecules are linked by two transformations: First changing yellow to magenta square and second changing light blue to dark blue circle. Nonadditivity is calculated using the compounds’ activities pAct_1-4_

NA is calculated from so-called double-mutant or double-transformation cycles (DTC) [15]. These cycles consist of four molecules, which give rise to four MMPs, and are linked by two identical transformations (Fig. 1). The nonadditivity of the DTC is calculated based on the molecules’ individual activities. Would the transformation be perfectly additive, the difference in activities would result in a value of zero. However, a non-zero value does not necessarily indicate nonadditivity. Assuming that each measurement among these double mutants contains experimental uncertainty, the experimental noise might add up and result in false nonadditive cases. Therefore, it is critical to distinguish real NA from assay noise.

Extensive work on experimental uncertainty and NA has been carried out by Kramer et al. [6, 15, 34–36]. For homogeneous data an experimental uncertainty of 0.3 log units was established, while heterogeneous data has a higher experimental uncertainty of 0.5 log units. In their publications regarding NA they created the statistical framework to systematically analyze NA. Kramer first developed a general metric and afterwards created an open-source python code to quantify NA, available on GitHub [6].

Despite the clear need for NA analysis it is generally not incorporated in classical QSAR applications and publications. NA clearly creates difficulties for linear SAR analysis approaches, such as standard MMP and FW analysis. These classical QSAR models will not work if the effect of introducing group R1 in the molecule is influenced by R2 or R3 [4].

Apart from classical CADD approaches, many machine learning (ML) and deep learning (DL) techniques became popular and are applied to a diverse range of questions—from generation of new molecules [37–40], to predicting binding affinities [41–49] and retrosynthesis predictions [50–53]. As shown recently by Sheridan et al. activity cliffs are a problem for QSAR models and are limiting their predictivity [54]. Thus, the question arises: How much are those methods influenced by NA? When activity data is used for the model training, NA might cause problems that are currently not considered adequately.

In this work we show a systematic analysis of AZ inhouse and public ChEMBL physicochemical and biological data with the aim to quantify and compare NA in assays and compounds in public and inhouse data. Nonlinear events occur in 57.8% of all the AZ inhouse and in 30.3% of all public assays, indicating the need for constantly integrating NA analysis in drug discovery projects and understanding the structural reasons behind it. Additionally, we trained ML models to evaluate the predictability of nonadditive data and could show their poor performance in all trained models.

## Methods

### NA analysis code

The open-source NA analysis code provided by Christian Kramer was used in this study (available on GitHub: https://github.com/KramerChristian/NonadditivityAnalysis) [6]. The code is written in Python making use of the cheminformatics libraries RDKit [55] as well as Pandas and NumPy. NA calculations are based on MMP analysis (upon the assembly of double-transformation cycles (DTC)), using an open-source code developed by Dalke et al., [56] which is an implementation of the MMPA algorithm by Hussain and Rea [3]. DTCs are assembled from four molecules, forming four MMPs, which are connected by two identical chemical transformations. The number of DTCs assembled per test depends on the size of the test. Nonadditivity values are calculated as difference in logged biological activities of the four compounds assembling the DTC (pAct_1-4_):

$$ \Delta \Delta {\text{pAct}}~ = {\text{ }}\left( {{\text{pAct}}_{{\text{2}}}  - {\text{ pAct}}_{{\text{1}}} } \right)~ - {\text{ }}\left( {{\text{pAct}}_{{\text{3}}} ~{-}{\text{ pAct}}_{{\text{4}}} } \right) $$

Nonadditivity analysis is performed for each assay independently.

### Data sets

In this study both public and inhouse data are analyzed in order to compare the occurrence of NA. By understanding both types of data valuable information can be concluded for CADD projects.

#### ChEMBL data set

Assay data was downloaded from ChEMBL version 25 (accessed Feb. 6, 2020) [57]. A ChEMBL target confidence score of at least 4 (confidence range from 0 to 9 based on available target information) was set as a threshold, resulting in 15,504,603 values.

#### AstraZeneca inhouse data set

All assays with an existing target gene ID were extracted from the internal AZ screening and test database (38,356 IT assays run from 2005 until 2020 across all AZ sites, accessed September 13, 2020).

#### Data curation

**Fig. 2:**
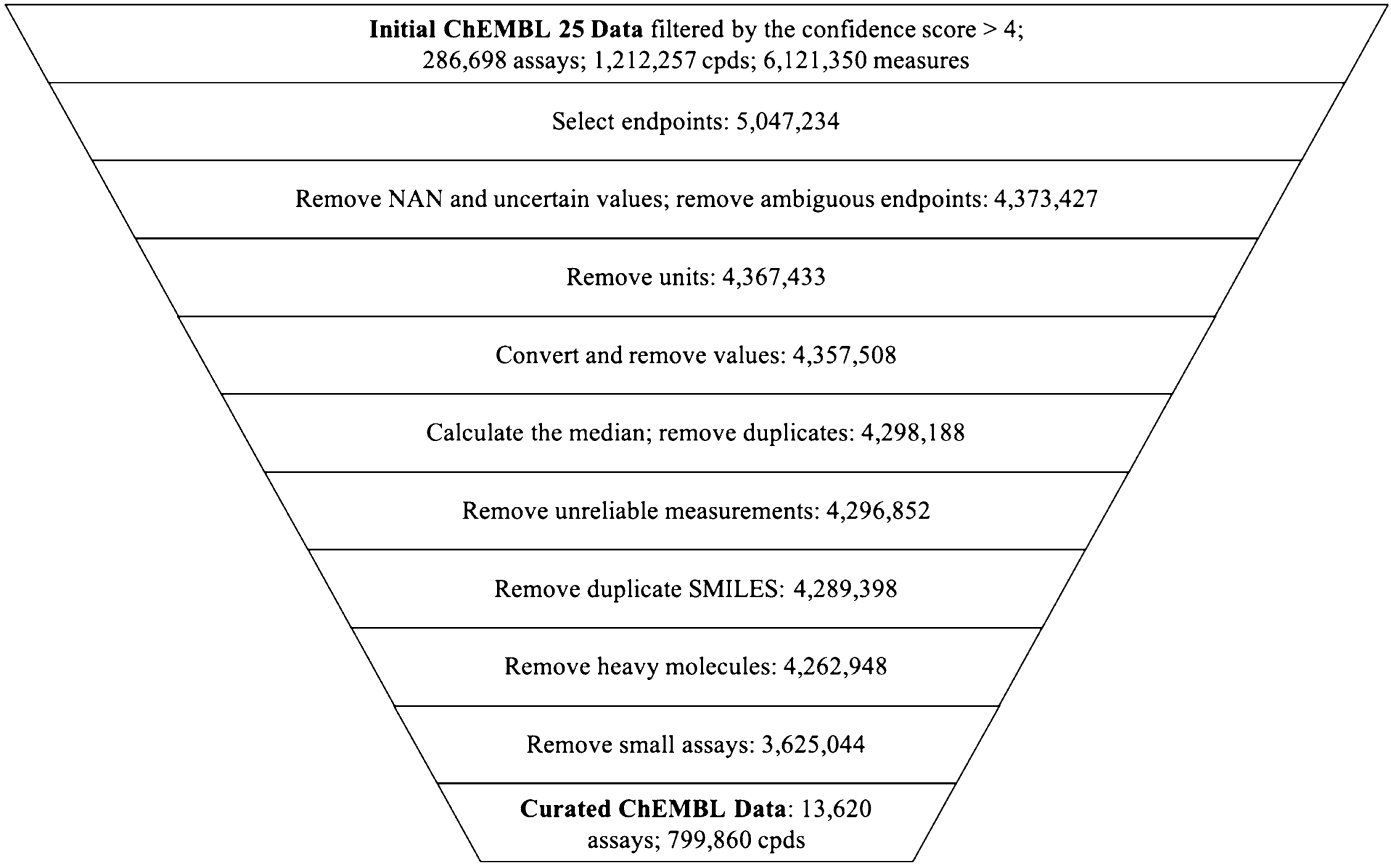
The data curation process of public ChEMBL25 data representing number of measurements after each cleaning step

Molecules were standardized with PipelinePilot (Additional file 1: Figure S1) including standardization of stereoisomers, neutralization of charges, and clearing of unknown stereoisomers. This step was followed by the enumeration of tautomeric forms and selecting the canonical tautomer with PipelinePilot. The same subsequent filtering steps were employed for both datasets using a Python script to make inhouse and public data comparable (Fig. 2). The filtering steps were the following: (1) All endpoints, suitable for NA analysis, were selected based on assay description. (2) Measurements without values as well as uncertain, i.e. qualified data with either “<” or “>” sign, and negative values were removed. (3) Only measurements with a defined unit (M, mM, μM, nM, pM, or fM) were kept. (4) The activity values were converted to the negative logarithm of the activity—pActivity (pAct) and unrealistic values, i.e. lower than 10 pM or higher than 10 mM, were discarded. Cases where the measurement was given as pActivity (e.g*.* pIC_50_) but had an indicated unit were discarded. (5) All compounds with multiple measurements in one assay, where the difference between the minimum and the maximum measurement was larger than 2.5 log units, were removed. For those kept, the median of the logged activity values was calculated. Only compounds with large measurement differences were removed, the assay itself was kept. (6) All compounds with different IDs and the same simplified molecular-input line-entry system (SMILES) strings were filtered out and only the compound with the highest activity value was kept. (7) The molecular size was restricted to 70 heavy atoms (atomic number > 1). (8) Last, small assays with less than 25 compounds were removed.

#### Data selection for QSAR models

The data sets for ML study were extracted from ChEMBL (Table 1). Public assays were chosen from the NA analysis of the ChEMBL data set that had (1) NA output, (2) >200 compounds, (3) >25 double-transformation cycles (DTC) per assay in order to observe the effect of NA on ML model performance.

**Table 1:** Description of ChEMBL assays selected for QSAR models

ChEMBL assay ID	# Cpds	# Cpds with significant NA (%)	# DTC	# unique cpds in DTC	# DTC with significant NA (%)	ChEMBL Version (access date)
1613777	3497	153 (4.38)	4333	1261	867 (20.01)	26 (06/20/2020)
1613797	6219	64 (1.03)	4523	701	694 (15.34)	27 (08/26/2020)
1614027	2876	76 (2.64)	4086	941	486 (11.89)	27 (08/26/2020)

Data curation was conducted with the Jupyter notebook (available on https://github.com/MolecularAI/NonadditivityAnalysis) and molecules were standardized with the included RDKit standardization code.

Each assay file contains: Compound IDs, SMILES, pActivity values, number of occurrences in DTCs, and an absolute NA value per compound (Additional files 2, 3, 4). An NA value above 1.0 log unit is considered to be significant, since this is double the expected experimental uncertainty for heterogeneous data. Additionally, a difference larger than 1.0 log unit indicates a divergence from perfect additivity by more than 10-fold.

### QSAR model building with Optuna

In order to build ML models, an automatic extensive hyper-parameter optimization tool, Optuna [58], was employed for each of the three selected ChEMBL data sets separately. Herein the optimization strategy is based on surrogate models, which is supposed to be superior to random or grid search. In order to analyze the effect of NA on ML performance Random Forest models were trained. In addition, a linear model (partial least square-PLS) was chosen as a base-line and is expected to perform worse for non-linear relationships than the RF model. RF is often considered as a base-line algorithm, being robust against over-fitting, while SVMs often push performance a bit further than RF [59]. The linear PLS model and a nonlinear SVM model using the default radial basis function (RBF) kernel were trained for one of the selected ChEMBL data sets (ChEMBL1614027) to assess their relative performance to the RF models. All models were trained using the scikit-learn framework [60].

The models are trained to predict the compounds’ pIC_50_ value of the selected data sets. This problem is often tackled using a binary classification into active/inactive compounds. However, the underlying problem is a regression and thus regression models were used for prediction of pIC_50_ values. In addition, the data has been binarized (based on a threshold of 5 for the pIC50 response value) to assess general model performance in a classification scenario. For all models 500 trial runs were performed using a 5-fold cross-validation to avoid overfitting. We used ECFP6 counts (as implemented in REINVENT [39]), which is a circular fingerprint with radius 3. This type of fingerprint captures the circular neighborhood of an atom and thus represent the presence of certain substructures. Using counts enables capturing the number of times the substructure is present in a molecule. The reported metrics for the regressors are R^2^ and RMSE as implemented in scikit learn.

#### Model training protocol

The following protocol was applied to ChEMBL data for training RF, SVM and PLS models. Herein, additive data refers to those compounds that had NA below the experimental uncertainty cut-off of 1.0 log unit and were thus not significant.

Models were trained based on different data selection strategies. First, compounds were considered that occur in DTCs (‘DTC-split’). For those compounds, their NA value is known and they can be classified as either additive or nonadditive. Second, we formed a data set based on all compounds (‘all-split’), in which compounds that are not in DTCs are assumed to be additive. For the first two selections further separation into training and test data is based on stratified splitting with 80% training and 20% testing, herein, only additive data is used for training, while different test sets are compiled consisting of additive or nonadditive data. Third, a splitting strategy was applied to construct the training data consisting of A or B compounds, while the testing data contained AB compounds (‘A-B-AB-split’). For this third set the information from the DTCs was leveraged to assign compounds to either training or test set. This splitting strategy was once applied using DTC data only and once adding those compounds, for which no DTC information is available. Due to a random starting point of assigning compounds to the additive test set, this strategy was performed twice using two different random seeds (4 and 7) in order to exclude the starting point being responsible for the performance of the model. For further information on the selection see Additional file 1 ‘A-B-AB splitting strategy’.

For all three data splits the following model training and testing strategy was applied:

(1.1) Optimization of hyper-parameters based on the training set (80% additive observations) with 5-fold cross-validation (i.e. mean performance of 5 models trained on 80% of the training set).
(1.2) Train final model on all of the training set using the best hyper-parameters from (1.1).
(1.3) Prediction of test sets
  *DTC-split* and *all-split*: predict two test sets – the non-significant test (20%), i.e. additive data only and the significant hold-out sets (all significant observations), i.e. nonadditive data only.
  *A-B-AB-split*: predict the non-significant AB test (20%), the significant AB test set, all remaining significant compounds not assigned as AB, and (if all data considered) the non-significant test (20%). Three tests sets are used for DTC data, four test sets for all data.
(1.4) Use R^2^ and RMSE to quantify performance.

#### Binary classification

(2.1) The predictions from (1.3) were dichotomized (threshold based on pActivity: 0 if pActivity < 5, 1 if pActivity > 5) and then compared to the true class (same threshold).
(2.2) Matthews correlation coefficient (MCC from scikit learn) is used to quantify performance. MCC is used due to several advantages for binary classification problems [61]: The MCC score is guaranteed to be between − 1 (anti-correlation) and 1 (perfect correlation), with 0 being the worst possible score, i.e. random. It takes into account the complete confusion matrix and thus provides a better balance between the different categories.

#### "Mixin" models

The effect of NA data during training and on the model performance on the test data was analyzed by adding increasing fractions of NA observations in the respective training sets (see Results). Therefore, we have trained models as described above and investigated whether the model performance changes by analyzing MCC values and confusion matrices. We used the hyper-parameters established earlier for the respective datasets.

Overall, for each selected ChEMBL data set 12 RF models were trained (Additional file 1: Table S1). For ChEMBL1614027 a PLS and SVM approach was trained additionally for the *DTC-split*.

## Results

The curated ChEMBL dataset contains 13,620 unique assays, 799,860 unique compounds and in total 3,625,044 measurements (Fig. 2), while the AZ inhouse data set consists of 6277 unique assays, 1,232,555 unique compounds and in total 5,801,969 measurements.

Most compounds (85%) in AZ assays have been measured more than once (Table 2), which is not the case for ChEMBL data (5%). This must be considered during the differentiation of true NA from experimental noise. It is, indeed, easy to detect strong NA, although weak NA can be easily confused with the experimental uncertainty. On the other hand, if the experimental noise is overestimated, potentially significant cases will be ignored and not considered for compound optimization. Therefore, it is critical to set the right threshold for experimental noise, since as mentioned before, it impacts the NA value twice as much as an individual biological measurement. Considering our data and the studies carried out by Kramer *et al.* regarding experimental uncertainty of public and inhouse data sets [34–36] 0.3 and 0.5 log units were used as thresholds for AZ and ChEMBL data respectively. Consequently, the NA values above 0.6 (AZ) and 1.0 (ChEMBL) log units were considered significant.

**Table 2:** The numbers describing both curated AZ inhouse and ChEMBL datasets along with the output of NA analysis

	AZ	ChEMBL
Nof		
Measurements	5,801,969	3,625,044
Cpds measured more than once (%)	85.8%	5.1%
Curated assays	6277	13,620
Unique cpds	1,232,555	799,860
Assays with NA	4030	7534
Assays with significant NA	3628 (57.8%)	4128 (30.3%)
Assays with NA*	3081 (49%)	–
Assays with strong NA#	1509 (24%)	1237 (9.1%)
Unique cpds showing significant NA*	114,862 (9.4%)	40,798 (5.1%)
Unique cpds showing strong NA#	5767 (0.5%)	8572 (1.1%)
Median nof		
Unique cpds per assay	233	35
Unique cpds per assay with NA output	490	39
DTC per assay with NA output	63	13
Unique cpds per assay with significant NA*	562.5	43
DTC per assay with significant NA*	88.5	23
Unique cpds per assay with NA*	662	–
DTC per assay with NA*	133	–
Unique cpds per assay with strong NA#	1093	52
DTC per assay with strong NA#	423	43

Nof: number of, cpds: compounds, DTC: double-transformation cycles

^*^ Significant NA: 0.6 log units for AZ inhouse data, 1.0 log units for ChEMBL data

^#^ Strong NA: > 2.0 log units

### Nonadditivity analysis

**Fig. 3:**
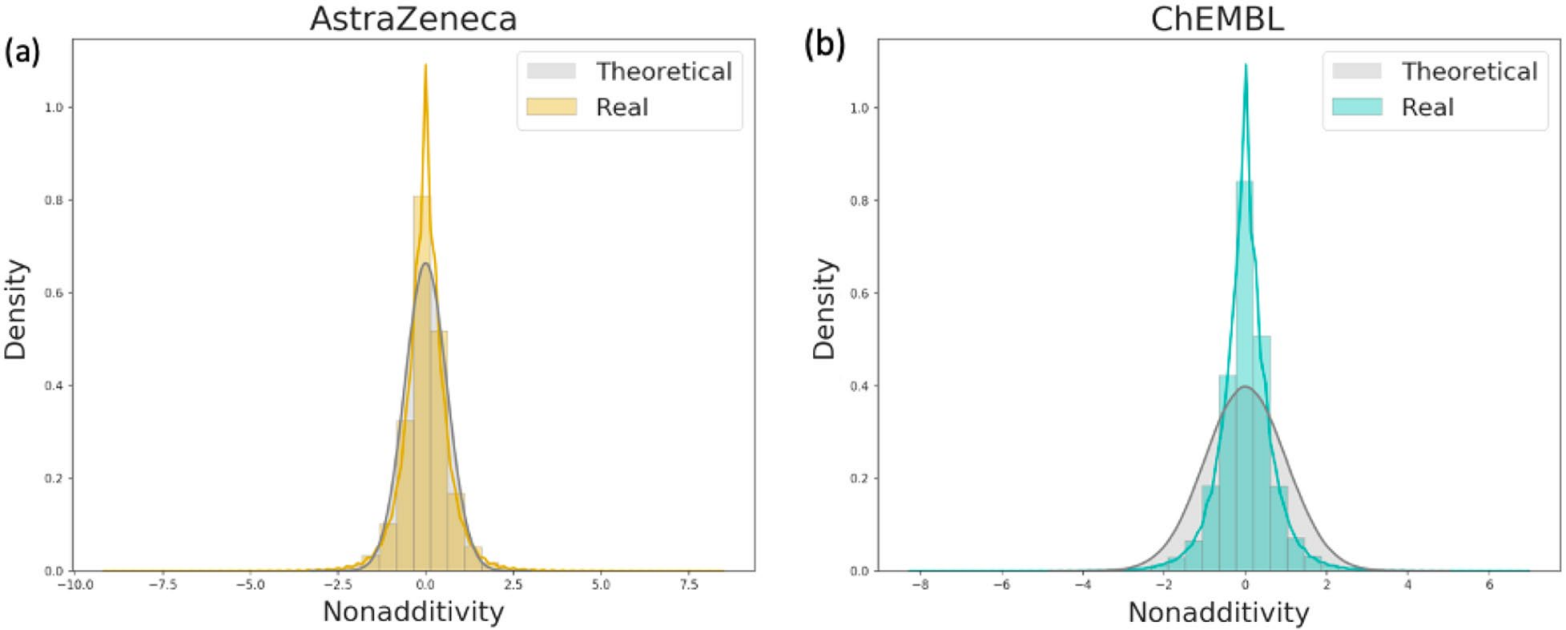
Theoretical NA distribution expected from an experimental uncertainty of **a** 0.3 and **b** 0.5 log units (grey lines), and observed NA distribution for all **a** AZ (yellow) and **b** ChEMBL (blue) assays, density = normalized count so that the area sums to 1

Figure 3 shows all observed NA of both AZ inhouse and ChEMBL data sets. The sign of the NA value depends on the order of the molecules within the double-transformation cycles (DTCs). Consequently, the raw data obtained after running the NA analysis contains both positive and negative values (Fig. 3). Negative values have afterwards been converted to absolute values. Most of the NA cases can be explained with the experimental noise (Fig. 3). Especially the major peak in the AZ and ChEMBL data are fully covered by the normal distribution expected from 0.3 and 0.5 log units of the experimental uncertainty respectively. A significant amount of DTCs not explainable by experimental uncertainty can be identified from the tail distributions.

According to Fig. 3 both AZ and ChEMBL NA distributions seem normal. However, the kurtosis, which is a measure of ‘tailedness’, is significantly large in both datasets (Table 3) and both fail the Kolmogorov-Smirnov [62, 63] tests for normality. Both AZ inhouse and public output of NA analysis is similar, yet undersampled in case of ChEMBL. Importantly, with the selected cutoff for experimental uncertainty of 0.5 based on previous analysis by Kramer et al. [6, 15, 34–36], NA events occur less often in public data than in inhouse data. Based on this, one might assume that nonlinear events are rare in public data and can be disregarded. However, the pattern of nonlinear observations in AZ data sets suggests that it must be considered more carefully and structural reasons must be thoroughly investigated since they might be hinting towards important structural features.

**Table 3:** Descriptive statistics of NA distribution in AZ inhouse and ChEMBL data sets

	Observations	Mean	Variance	Std	Skewness	Kurtosis
AstraZeneca	3,053,055	0	0.42	0.65	0	3.13
ChEMBL	1,246,975	0	0.46	0.68	0.01	4.52

Note that all NA values have not been converted to absolute values prior to these calculations

In order to compare the distribution of NA in two groups, two tests have been performed: (1) Kruskal-Wallis H Test [64], that does not have the assumption of normality, testing the null hypothesis that the population median of both of the groups is equal; (2) Mann-Whitney U tests [65] have been employed to test the null hypothesis that it is equally likely that a randomly selected measurement from one group of observations will be less than or greater than a randomly selected measurement from the second group of observations. According to the obtained results from both tests, the NA value distribution in AZ and ChEMBL data sets are not different from a given level of confidence (p-value = 0.07).

Importantly, public data has a larger number of assays with fewer measurements and unique compounds (Table 2). The number of assays showing significant NA in ChEMBL data is lower (30.3%, higher than 1 log unit) than in AZ inhouse data (57.8%, higher than 0.6 log units). However, ChEMBL assays, in general, contain fewer compounds, therefore the number of DTCs and hence the chance of a strong NA occurring is lower.

**Fig. 4:**
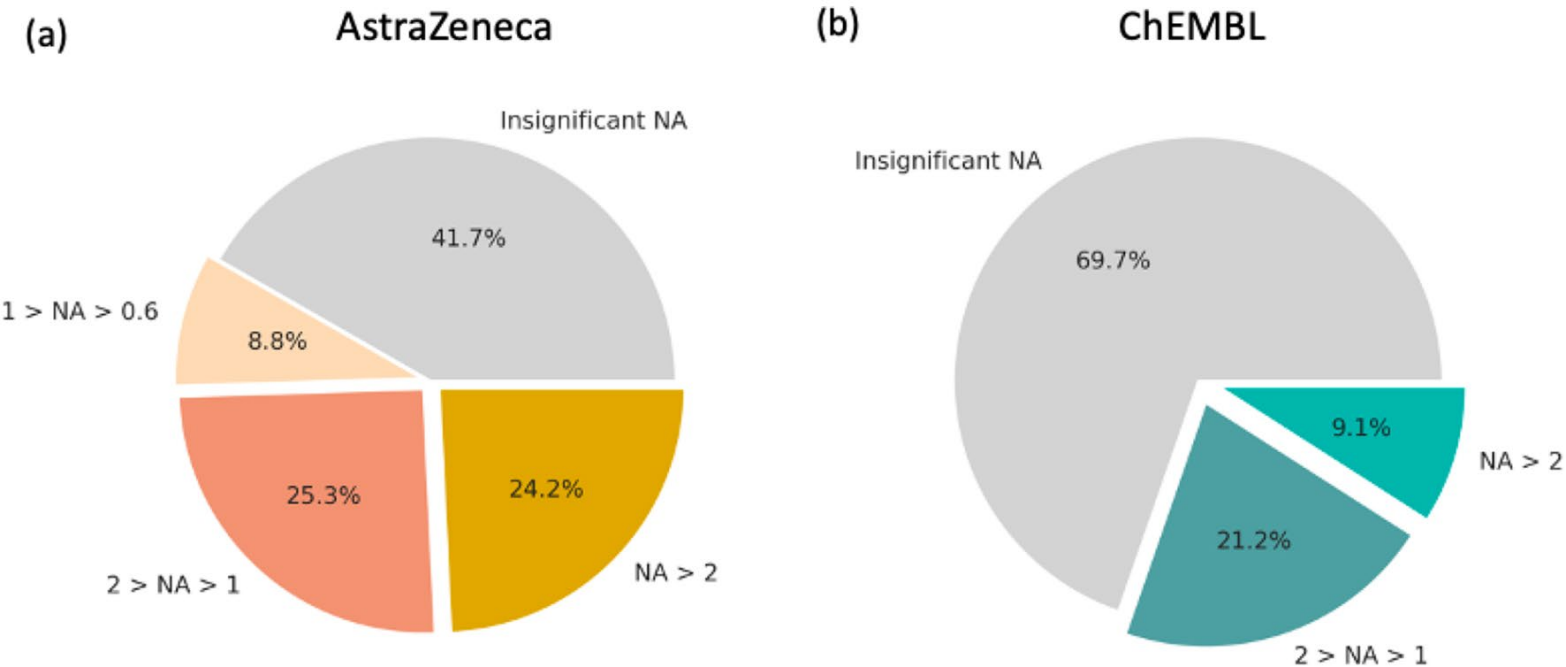
NA distribution among all curated assays from AZ inhouse (**a**) and public ChEMBL (**b**) data sets

Less than half of the assays (41.7%) in AZ screening and test database are either additive or no DTCs were assembled (Fig. 4a). This number is higher in public bioactivity data (69.7%, Fig. 4b), which can be explained by the higher threshold of experimental noise and smaller assay sizes. Remarkably, 24% of all AZ inhouse assays show strong NA (above 2 log units), whereas in ChEMBL bioactivity data strong NA is observed in 9.1% of all assays. Yet, various virtual screening studies depend on public datasets and it is crucial to take NA into account whilst judging the performance of predictive models since 1 out of 10 assays might not be additive.

**Fig. 5:**
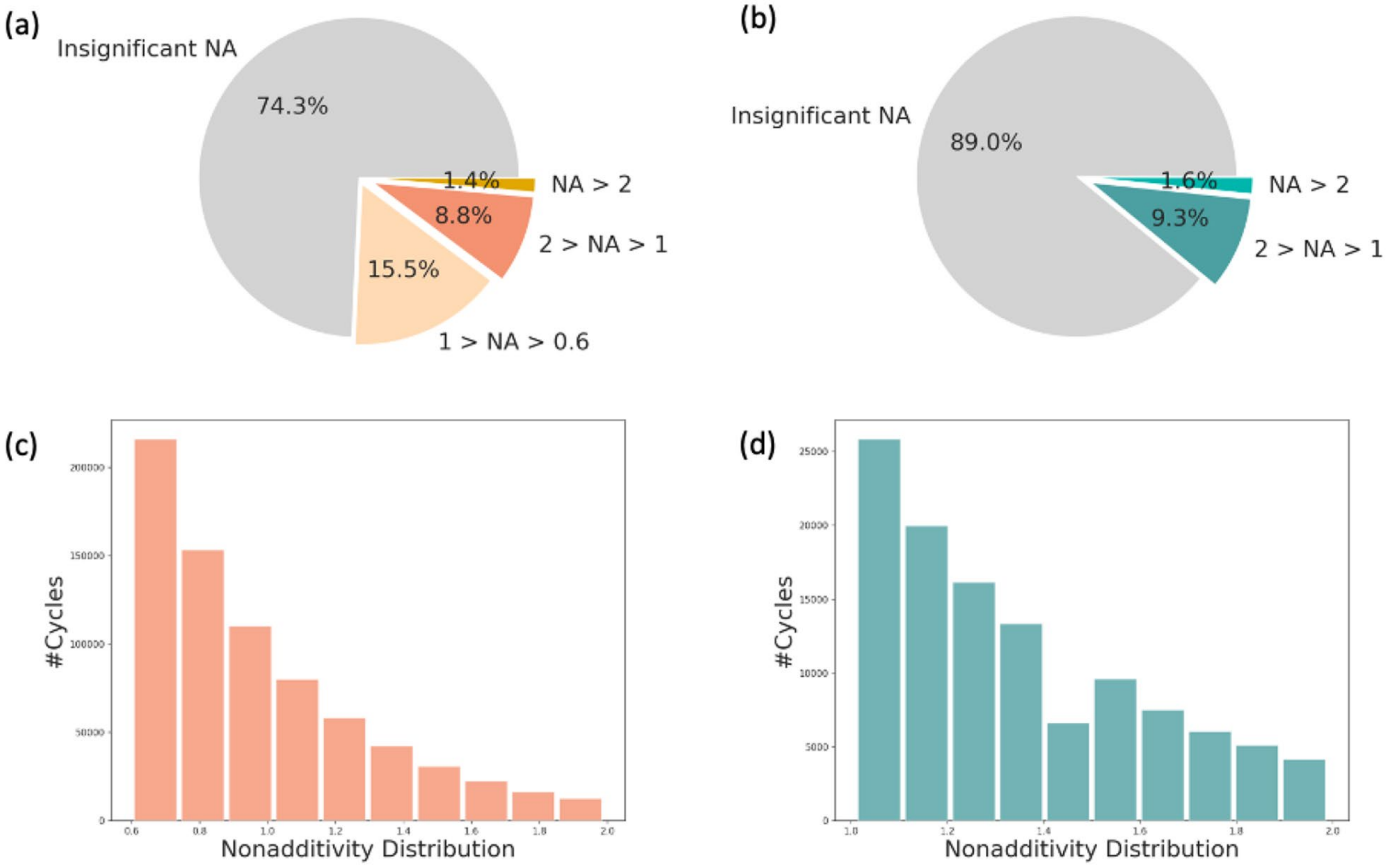
NA distribution for all DTCs among curated assays from AZ (**a**) and ChEMBL (**b**) data sets. **c** NA distribution of DTCs showing significant NA score (from 0.6 to 2 log units) in AZ (**c**) and (from 1 to 2 log units) ChEMBL (**b**) bioactivity data

Besides the number of assays, NA can also be analyzed for DTCs. On average one out of four and one out of ten DTCs is not additive for AZ inhouse and ChEMBL data respectively (Fig. 5a and b). The distribution of NA among DTCs shows significant NA up to 2 log units indicating a gradual decrease in the number of cycles with the increasing NA value (Fig. 5c and d).

**Fig. 6:**
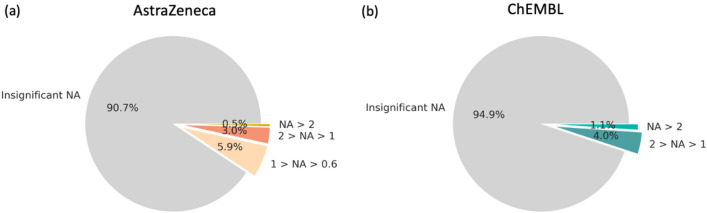
NA distribution among all unique compounds from AZ (**a**) and ChEMBL (**b**) data sets

Out of all compounds 9.4% from AZ and 5.1% from ChEMBL data sets show a significant NA shift (Fig. 6). As mentioned before, assay sizes and different thresholds for the experimental uncertainty influence these numbers.

**Fig. 7:**
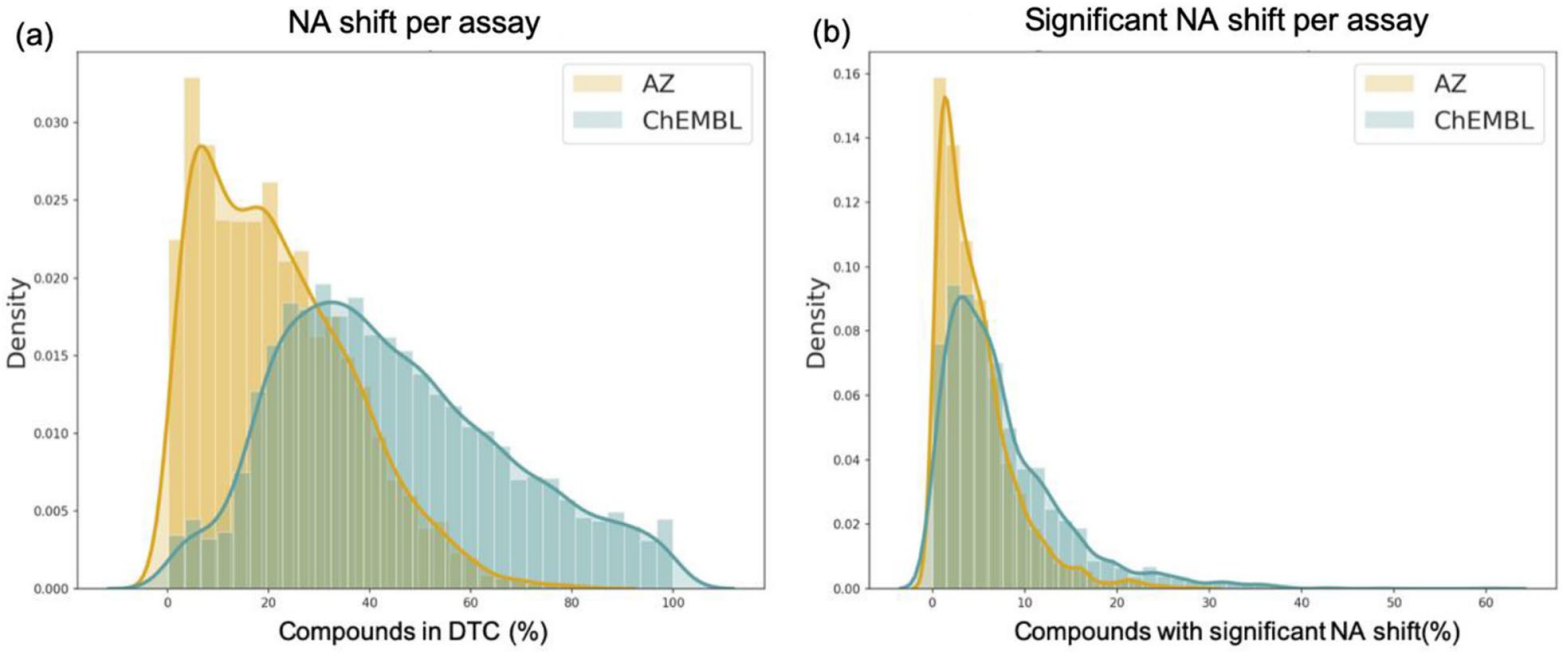
**a** Distribution of the compounds in DTC. **b** Distribution of the compounds showing a significant NA shift per assay, density = normalized count so that the area sums to 1

Bioactivity assays from ChEMBL have a smaller number of compounds and a lower number of DTCs per assay. Yet, Fig. 7a and b show the shifted distribution of the compounds occurring in double-transformation cycles per assay. Surprisingly, there are more than a hundred assays in public data sets in which almost all compounds participate in the assembly of DTCs. This might be due to very small structural variations of tested molecules. AZ inhouse assays tend to be more diverse. Ultimately, testing more compounds results in a lower percentage of unique molecules showing NA. Even though the median number of DTCs is higher in AZ assays, the number of compounds tested in these data sets is also larger, resulting in a relatively lower ratio.

**Fig. 8:**
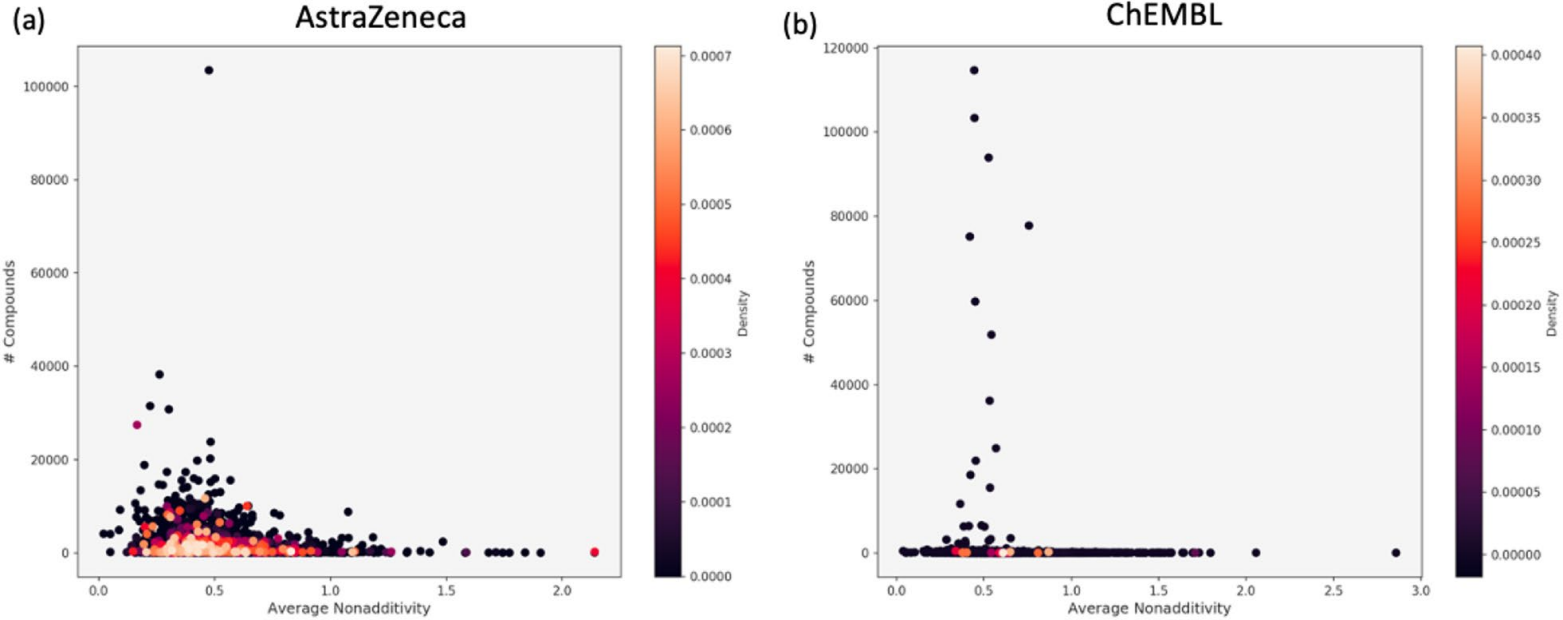
Density distribution of the assays showing significant NA from AZ (**a**) and ChEMBL (**b**) based on the average NA and the number of compounds in each assay

NA distribution according to the number of compounds in assays (Fig. 8) indicates that most of the assays in the AZ database contain up to 20,000 compounds and generally smaller assays show higher NA. On average, ChEMBL assays are smaller (Table 2), although several large assays vary in size resulting in a more spread out pattern (Fig. 8). Herein, highest NA values occur in both small as well as large assays (Additional file 1: Figure S2). Furthermore, the density distribution of all assays shows the assembly around the experimental uncertainty.

**Fig. 9:**
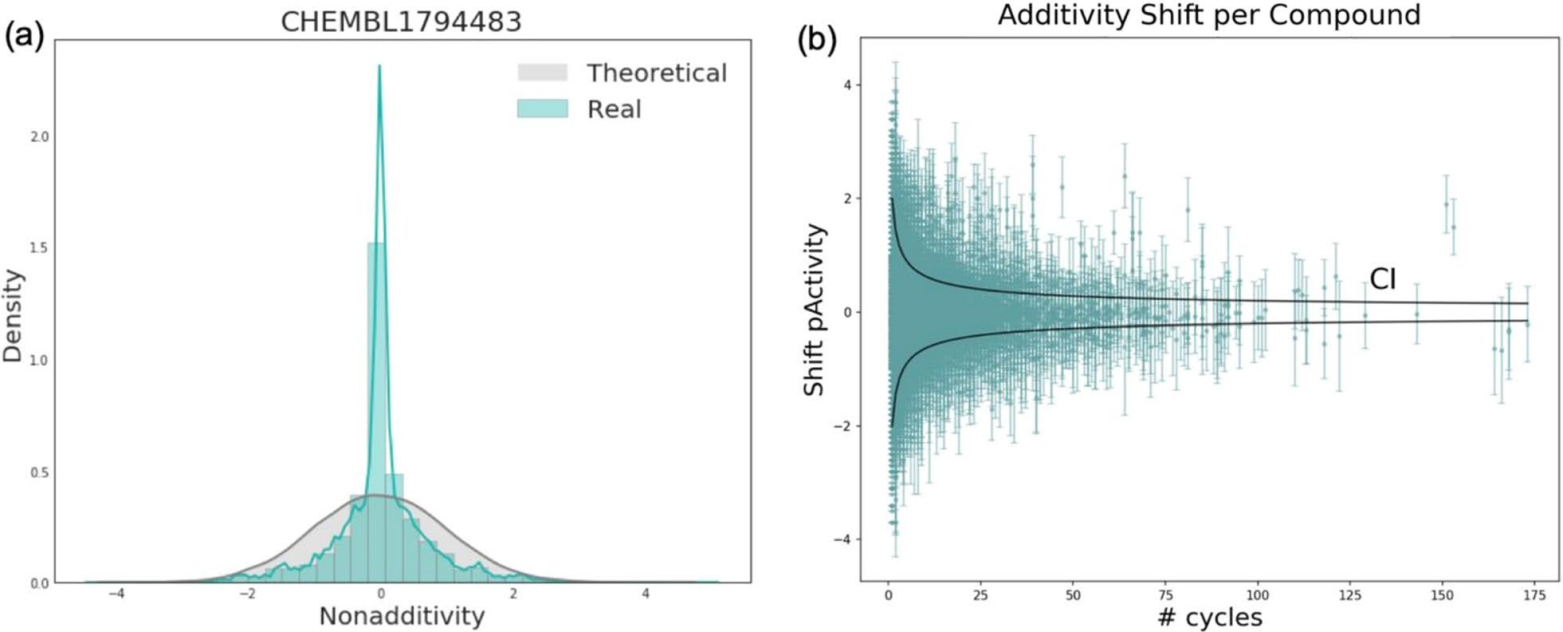
**a** Theoretical NA distribution expected from an experimental uncertainty of 0.5 log units (grey line), and an actual NA distribution for CHEMBL1794483 assay (blue), density = normalized count so that the area sums to 1. **b** The average additivity shifts per compound and the standard deviation of the shift for the CHEMBL1794483 assay. Black lines show the confidence interval (CI = 95%) indicating the area where the compounds should appear in case of additivity given the selected threshold of experimental uncertainty (0.5 log units in this case)

CHEMBL1794483 is the largest bioassay obtained from CHEMBL25 (Additional file 1: Figure S2). Initial data of the quantitative high throughput screening for the inhibitors of polymerase Iota contains 115,311 measurements, 33,777 DTCs have been assembled with an average NA score of 0.44. The NA distribution is almost entirely covered by the theoretical normal distribution expected from the experimental noise of 0.5 log units (Fig. 9a). The assembled DTCs contain 24,238 compounds and the average additivity shift for each compound is depicted in Fig. 9b. In general, it is impossible to point out which molecule causes the NA in a given DTC without further structural information. If the compound occurs in many DTCs with high average NA shift (always with significantly low or high potency), it indicates either a plain error, i.e. a wrong measurement, or structural properties that drastically increase or decrease the compound’s biological activity.

**Fig. 10:**
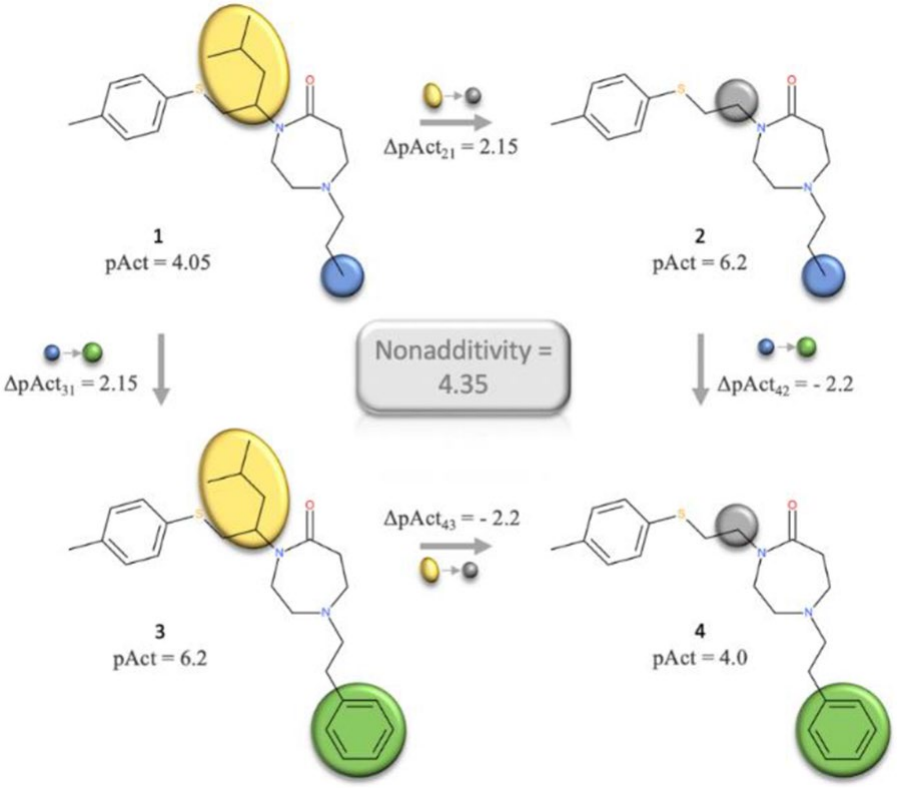
The DTC from CHEMBL1794483 assay with one of the highest NA score (4.35)

Figure 10 shows the DTC from CHEMBL1794483 assay with one of the highest NA scores. If the SAR was perfectly additive then the removal of isopropyl group and attaching the benzyl group should have resulted in a significant increase of the potency, yielding pActivity of 8.35. Instead, the activity of the fourth compound even decreased and is lower than compound 1.

### QSAR model evaluation

In the second part of the results, the influence of NA on ML performance will be analyzed. Herein, three different ChEMBL assays (Table 1, Additional file 1: Figure S3) were used to analyze the following aspects: (1) Can NA compounds be correctly predicted from a model based on additive data? (2) Does the integration of NA data into training increase model performance?

**Fig. 11:**
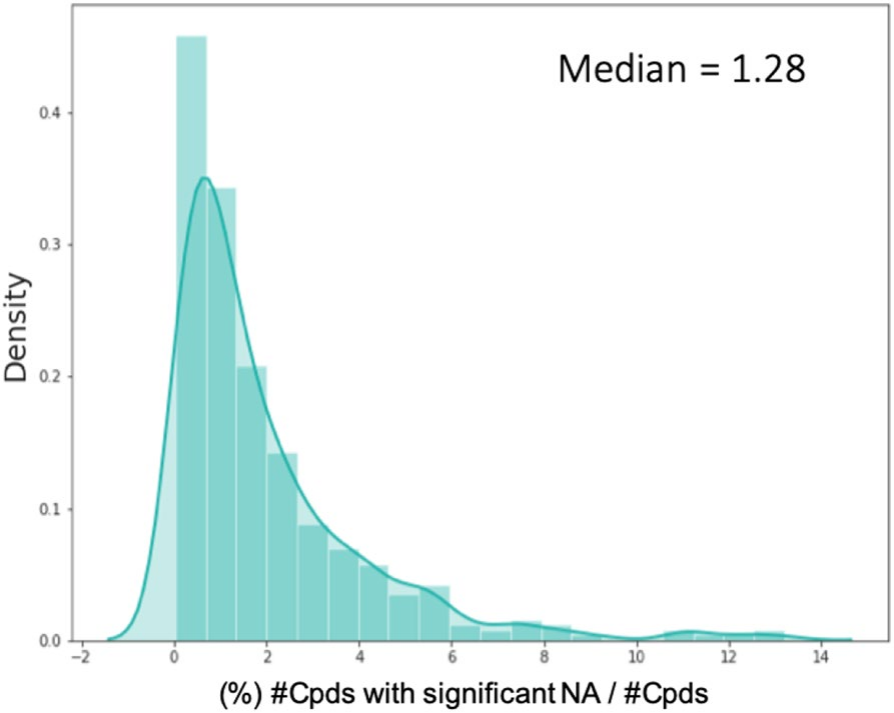
Distribution of NA compounds (%) and the number of DTCs (%) in ChEMBL assay that show NA, density = normalized count so that the area sums to 1

The data sets for the second question were constructed based on the median number of compounds with NA observations (Fig. 11). Thus, three sets were constructed for each ChEMBL assay containing Q1 (0.6%), median (1.3%) and Q3 (2.6%) of NA compounds. The NA compounds were selected using a stratified split. The NA hold-out set was constructed form the Q3 (2.6%) split, i.e. all models were evaluated on the same subset of observations to ensure comparability of performance.

**Fig. 12:**
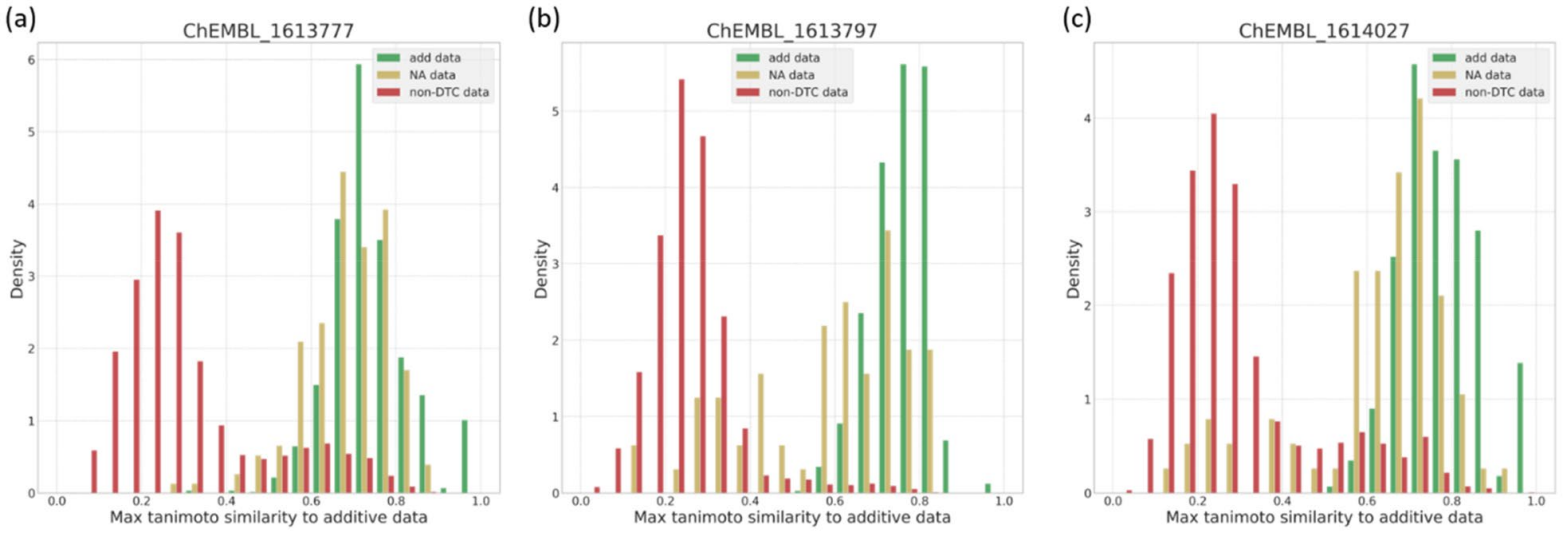
Overlay of tanimoto similarity distributions for additive data (green), nonadditive data (yellow) and non-DTC data (red). For each compound the maximum Tanimoto similarity to any other compound was calculated using ECFP6, excluding its identity. For both other data sets, i.e. nonadditive and non-DTC data, the similarity was calculated against the additive data, density = normalized count so that the area sums to 1

In order to check that any difference in performance is not purely due to a different biological/chemical space, two aspects were checked: (1) the coverage of pIC50 values between additive and nonadditive (Additional file 1: Figure S3) and (2) the similarity between the compounds (Fig. 12). The similarity of nonadditive and additive compounds, measured by tanimoto similarity using ECFP6, overlaps well, which would be expected, since they are related by MMPs. However, the remaining assay data, where no DTC can be constructed, is significantly different from the additive data. The range of pIC_50_ overlaps well for all three data sets in all three assays.

#### DTC-split and all-split model performance

Based on the automatic hyper-parameter training using Optuna, individual RF models were generated for each of the three selected ChEMBL assays (Additional file 1: Tables S2–S4). Additionally, a linear model (PLS) and a SVM was trained for ChEMBL1614027 (Table 4 and Additional file 1: Table S2). The model performance metrics show that the RF model build using DTC-split performs best. Herein, the R^2^ for the cross-validation on the training data is significantly better for RF with the lowest RMSE. The performance on the additive test set differs only slightly between all three models. The good performance of PLS in this case can be explained with the additivity of this test data, thus also a linear model would be expected to perform well. All models are significantly worse for the nonadditive test data, with PLS being the worst. A binary classification after prediction of the pIC_50_ values results in a minor improvement of MCC for SVM compared to the RF model. Interestingly, the overall performance for both training and additive test set decreases when all data (all-split) is used for training the models. For the NA set only a minor improvement in RMSE values can be observed, while overall the model is still non-predictive for this data set, i.e. negative R^2^ and an RMSE > 1.2.

**Table 4:** Model performance measures for ChEMBL1614027 based on DTC-split model 1 and all-split model 5

	RF					SVM					PLS		
	Train R^2^ (RMSE)	Test R^2^ (RMSE)		Test MCC		Train R^2^ (RMSE)	Test R^2^ (RMSE)		Test MCC		Train R^2^ (RMSE)	Test R^2^ (RMSE)	
		A*	NA^#^	A*	NA^#^		A*	NA^#^	A*	NA^#^		A*	NA^#^
DTC-split	**0.93 (0.15)**	**0.60 (0.36)**	− 0.48 (1.26)	0.62	0.28	0.84 (0.23)	**0.60 (0.36)**	− 0.46 (1.25)	**0.69**	0.31	0.76 (0.28)	0.54 (0.39)	− 0.60 (1.31)
All-split	0.78 (0.33)	0.34 (0.57)	**− 0.35 (1.20)**	0.40	0.22	0.49 (0.50)	0.32 (0.57)	− 0.43 (1.23)	0.47	**0.32**	0.45 (0.52)	0.25 (0.60)	− 0.39 (1.22)

Bold values are best performance measures across DTC-split and All-split and across different ML approaches

Train R^2^ is based on 5-fold cross validation results

^*^Additive test data

^#^Nonadditive test data.

**Fig. 13:**
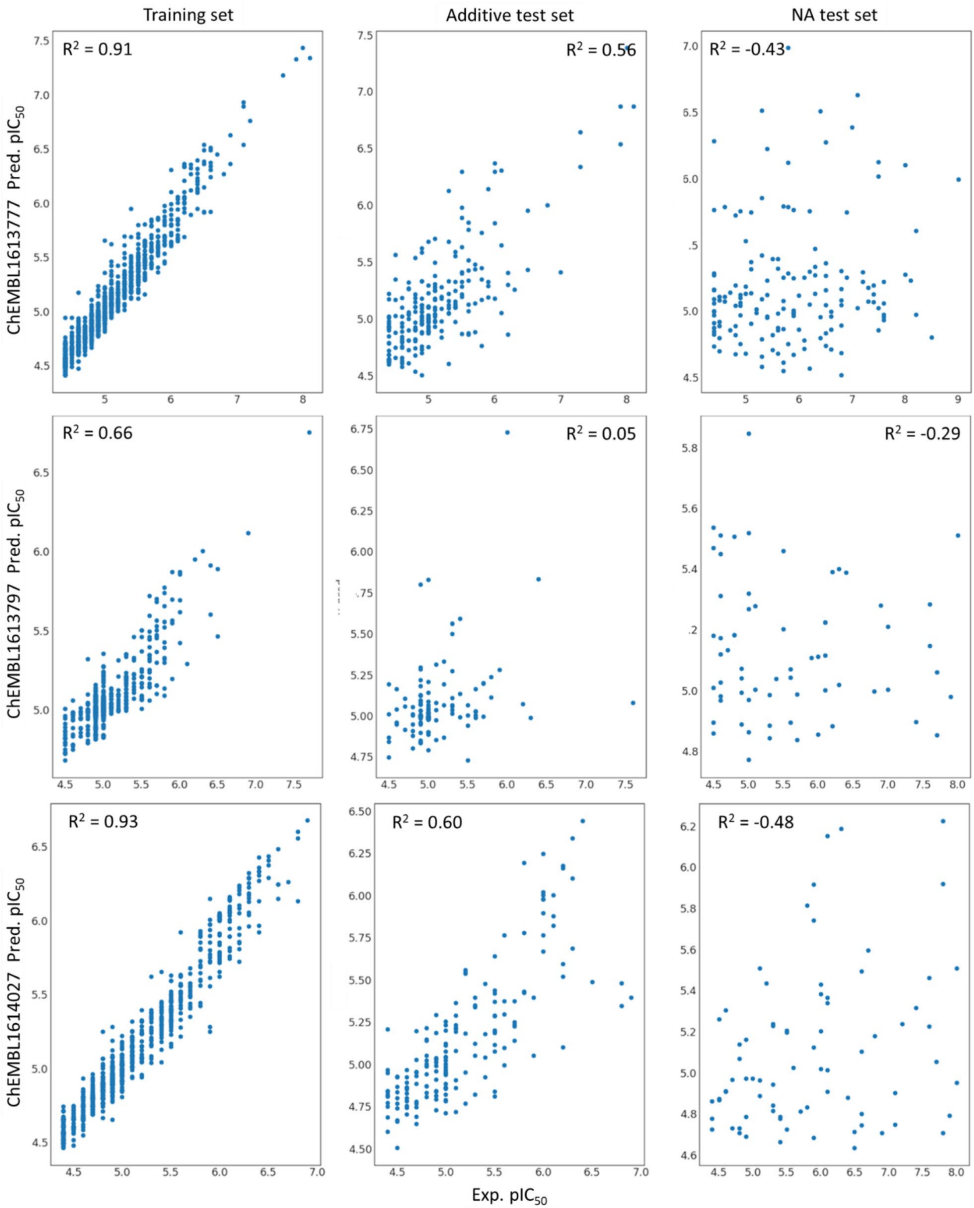
Correlation plots with RF predictions for all three ChEMBL assays based on DTC-split model 1

Both RF and SVM show similar test set performances for ChEMBL1614027, while SVM performance was more volatile to the actual choice of hyper-parameters (Table 4, Additional file 1: Figure S4). While the RF model built with DTC-split data for ChEMBL1614027 and ChEMBL1613777 performed well on training and additive test sets, it performed rather badly for ChEMBL1613797 with R^2^_train_ = 0.66 and R^2^_A-test_ = 0.05 (Fig. 13, Table 5), indicating that this set is very difficult to learn. Importantly, for all three assays the performance on NA test data consistently dropped. In addition to the drop in correlation between experimental and predicted data the predicted error (RMSE) increases for all NA data sets.

**Table 5:** RF model performance measures based on DTC-split model 1 and all-split model 5

ChEMBL data		Train R^2^ (RMSE)	Test R^2^ (RMSE)		Test MCC	
			A*	NA^#^	A*	NA^#^
1613777	DTC-split	**0.91 (0.17)**	**0.56 (0.44)**	− 0.43 (1.30)	**0.48**	**0.02**
	All-split	0.64 (0.47)	0.22 (0.68)	− **0.34 (1.25)**	0.34	0.00
1613797	DTC-split	**0.66 (0.22)**	**0.05 (0.41)**	− 0.29 (1.14)	**0.45**	− 0.03
	All-split	0.43 (0.45)	0.05 (0.58)	− 0.31 (1.11)	0.07	**0.00**

Bold values are best performance measures across DTC-split and All-split

Train R^2^ is based on 5-fold cross validation results

^*^Additive test data

^#^Nonadditive test data

The same drop in performance on the training and additive test sets when including all assay data (all-split) can be seen for ChEMBL1613777 but not for the already bad performing ChEMBL1613797 (Additional file 1: Tables S3 and S4).

Furthermore, a binary classification of the predicted values was done and the MCC was calculated as well as confusion matrices generated. Both show that it is much harder to accurately predict the NA test sets (Tables 4, 5, Additional file 1: Figure S5).

#### A-B-AB-split model performance

The hypothesis for splitting assay data into A-B-AB was that it might be easier to predict compounds, if they were not distributed randomly into test or training set, but by using the information from the DTCs, i.e. A and B contain information about both transformations for compound AB.

The splitting into different compound sets leveraging the information from DTCs resulted in an increased model performance for ChEMBL1614027 on the additive test set but a drop in performance for the NA test set in combination with an increased RMSE (Additional file 1: Table S2). This was observed for both DTC data only as well as models built with all assay data included. For ChEMBL1613777 the models performed similarly well on all test sets with DTC data only (Additional file 1: Table S3). Using all assay data, the model performance on the additive AB test sets increased significantly, while the performance for the NA test sets did not change. The already bad performing model for ChEMBL1613797 did not improve at all using the A-B-AB-split (Additional file 1: Table S4).

#### Mixin model performance

In a subsequent test, NA data was added to the training set to evaluate whether this could improve the prediction for NA data. For these "*mixin*" trials, it appears that for all ratios and all assays there is no significant difference in performance, neither for the performance on the predicted pIC_50_ values evaluated by R^2^ and RMSE nor for the binary classification evaluated by MCC (Table 6, Additional file 1: Tables S2–S4, Figure S6). This might be either because it is difficult learning from those examples or because they are too few in number in order to impact the performance significantly.

**Table 6:** Performance measures for binary classification of *mixin* models, Q refers to relative quantity of NA compounds added to the training data

ChEMBL data		RF (MCC for test)			
		Q0 (0.0%)^*^	Q1 (0.6%)^*^	Median (1.3%)^*^	Q3 (2.6%)^*^
1613777	DTC-split	0.02	0.04	− 0.04	0.02
	All-split	0.00	0.04	− 0.05	− 0.02
1613797	DTC-split	− 0.03	0.07	0.20	− 0.12
	All-split	0.00	0.00	0.00	0.00
1614027	DTC-split	0.28	0.28	0.28	0.20
	All-split	0.22	0.04	− 0.05	0.11

^*^ Test set size for Q0 differs from Q1/Median/Q3.

## Discussion

The project aimed to analyze the occurrence of NA in public and inhouse data and its influence on machine learning performance.

One of the biggest challenges during this process is the data pre-processing to make both sets comparable. Thus, additional cleaning steps were applied to ChEMBL bioactivity data, such as filtering by the target confidence score to increase the data reliability. The final ‘cleaned’ dataset depends on the experience and decision-making of the researcher to correctly choose which assays are compatible with the analysis.

The size restriction of the molecules was based on the structural transformations and similarities, the upper limit of the molecular size included and exchanged during the transformations must be set carefully. In this study, a maximum of 70 heavy atoms and the transformation of a maximum 1/3rd of the molecule were used. Without having these limitations, the following issues may arise: (1) large molecules, such as peptides are not compatible with the NA analysis since it is impossible to track small functional groups; (2) performing calculations on large molecules is computationally expensive; (3) cases where the functional group represents too large a proportion of the molecule will most likely result in NA since almost the whole compound is transformed and the corresponding binding mode is more likely to change.

In addition to the size restrictions of molecules, limiting assay size after all the data-cleaning steps is also crucial. On one hand, small assays should be discarded, because there is a lower probability of DTCs assembling. In this research project, 25 was set as the lowest number of unique compounds per assay. Since most of the assays are small (half of the measurements in both inhouse and public data sets were concentrated in a few hundred assays only), it also influences the general statistics resulting in no NA output. One might argue that the majority of the assays are additive, however, most of them are too small to draw any meaningful conclusions regarding their NA.

According to the results, significant nonlinearity occurs once in every second assay in AZ inhouse and once in every third biological and physico-chemical assays in ChEMBL databases. Importantly, significant nonadditive events are less frequent in public data sets. The reasons for this can be: (1) potential bias in reporting single series or positive SAR results; (2) the smaller size of public bioactivity assays, resulting in less DTCs; (3) a higher threshold of the experimental uncertainty for the entire data, as some assays have significantly higher experimental noise. An additional influence is the reliability of the compound measurements. Since in the inhouse database a majority of compounds is measured several times in each assay the measurements are more reliable. This is not the case in the public data sets, where only 5% of the compounds are measured more than once in each assay.

Prior to the analysis, it is crucial to carefully set the thresholds for the experimental noise to point out true NA cases. Strong NA stands out from the rest of the data and it is easy to spot, while weaker NA is usually blended with the experimental noise. As described by Kramer et al. [6]. NA analysis can estimate the upper limit of an experimental uncertainty for specific biochemical assays, which is crucial in differentiating true NA from the assay artefacts. However, it is less straightforward to select the threshold for large data. While experimental noise among most of the inhouse assays might be 0.2 log units, there are still some assays with larger errors. The problem with the higher limits of the experimental noise is the higher amount of insignificant NA cases. By choosing 0.5 log units for public data, we potentially cover all the assay artefacts, still, we might have ignored potentially true NA cases.

Based on three showcases, we elucidated the impact of NA data in QSAR models and how well NA compounds can be predicted by those models. Herein, ChEMBL1613777 and ChEMBL1614027 achieve good generalization during training the models as shown by high cross validation R^2^ values. ChEMBL1613797 assay data on the other hand proved to be difficult and the models do not generalize well. Thus, main conclusions are drawn from results based on ChEMBL1613777 and ChEMBL1614027. A clear trend for all three selected assays was a bad prediction of NA compounds independent of the models’ training performance. This observation remains true for different selection of training data, i.e. only based on compounds occurring in DTCs or based on all assay data. Employing a different splitting strategy (A-B-AB-split) by leveraging the information from DTCs resulted in a better performance for additive compounds but no or a slight drop in performance for NA compounds. The reason for this might be that the model learns the additivity from compounds A and B. Thus it can only predict compound AB correctly, if the additivity assumption holds true. If, however, compound AB displays an unexpected change in affinity, i.e. the compound is nonadditive, the model has even more problems predicting this compound compared to other models trained on data where the compounds were randomly assigned to either training or test set. Overall, this analysis shows how important a careful analysis of nonadditivity in data is. Even though the inclusion of those compounds does not affect the performance too much, nonadditive compounds cannot be predicted correctly and thus display a problem for QSAR models. Here, one also has to keep in mind that especially those NA compounds might have an interesting SAR that can be further leverage in the drug design process.

NA can be a problem for linear SAR techniques. Yet, if used intentionally, it can be an important tool for drug discovery. This study provides a detailed picture of the NA pattern amongst the inhouse and public databases, providing the global distribution of nonlinear events amongst assays and unique compounds. A careful understanding of the data is the key to successful decision-making. By conducting NA analysis one can easily identify outliers, detect potential assay artefacts, or key conformational changes. It is crucial to understand the possible experimental noise, that can be underlying most of NA cases. Therefore, one must always keep in mind the origin of a given assay, the reliability of the measurements, and a possible upper limit of experimental uncertainty.

By systematically incorporating the NA analysis into the drug discovery projects, detection of interesting interactions and key SAR features will be easier and will eventually provide more structural insights for rational drug design.

## Conclusions

Identifying NA in the SAR data sets can be crucial by suggesting important structural features for the compound optimization. However, nonadditive events can be caused by the random addition of experimental uncertainty, which is important to consider during the interpretation of results. The impact of the experimental noise increases with the size of the assay, as more double-transformation cycles can be assembled. NA analysis in the AZ compound database suggests that significant nonlinear events are more frequent in AZ inhouse data than public ChEMBL data. By considering only public data one might assume that NA is a rare event and important cases can be neglected. AZ data points out the fact that this is not true and the statistical framework of the NA analysis should be systematically implemented in SAR projects and discussed in publications for rational drug design.

Retrospectively, it is difficult to identify whether a specific change lead to a general increase or decrease in activity. From MMP studies we know that 100-fold improvements are very rare events of about 1% [66]. Our numbers (1–3%) suggest that electrostatic or steric problems occur more frequently than expected from SAR data because of the undersampling of negative data. This undersampling might be a reason why QSAR models have problems with describing activity cliffs despite being often based on non-linear algorithms. This would also be useful for setting a baseline of performance to be expected from such models.

Currently, the sign of a NA value does not provide valuable information since the order of compounds does not indicate the effect of a given transformations. In other words, one cannot establish which feature leads to the gain or loss of activity from investigating a specific double-transformation cycle. It would add another level of information to see the pattern of NA distribution in terms of boosting or decreasing the biological effect, whether the cases are equal or mostly lead to the loss of biological activity. For further follow-up work it would be of interest to draw conclusions about patterns in NA, i.e. if target-specific or non-target specific modification can be identified that always lead to NA, both on a per dataset basis and across public and inhouse data.

## Supplementary Information

**Additional file 1.** Additional figures.

**Additional file 2.** ChEMBL1613797 data set with obtained NA values for ML approach.

**Additional file 3.** ChEMBL1614027 data set with obtained NA values for ML approach.

**Additional file 4.** ChEMBL1613777 data set with obtained NA values for ML approach.

## Data Availability

The datasets supporting the conclusions of this article are included within the article and its additional files. S1: Additional figures. The Jupyter notebook for data preparation and NA analysis is available on GitHub (https://github.com/MolecularAI/NonadditivityAnalysis). ChEMBL data sets (ChEMBL1613777/1613797/1614027) with obtained NA values for ML approach are available as csv files. Each assay file contains: Compound IDs, SMILES, pActivity values, number of occurrences in DTCs, and an absolute NA value per compound. For compounds without number of occurrences in DTCs and an absolute NA value no DTC could be assembled during nonadditivity analysis. Nonadditivity analysis code was made available by Christian Kramer on GitHub (https://github.com/KramerChristian/NonadditivityAnalysis).

## Abbreviations

NAA: NA analysis
AZ: AstraZeneca
SAR: Structure-activity relationship
QSAR: Quantitative structure-activity relationship
AI: Artificial intelligence
ML: Machine learning
FW: Free-Wilson
MMPA: Matched molecular pair analysis
FBDD: Fragment-based drug discovery
CADD: Computer-aided drug design
SMILES: Simplified molecular-input line-entry system
SBDD: Structure-based drug design
RF: Random forest
SVM: Support vector machine
