# Supplementary material for: Nonadditivity in public and inhouse data: implications for drug design
Source: J Cheminform. 2021 Jul 2;13:47. doi: 10.1186/s13321-021-00525-z (PMC8254291; doi:10.1186/s13321-021-00525-z)
Supplement: Supplementary file 1 — Additional file 1. Additional figures. [file 13321_2021_525_MOESM1_ESM.pdf]

SUPPORTING INFORMATION

Nonadditivity in Public and Inhouse Data – Implications for Drug Design

D. Gogishvili[1,#,¤], E. Nittinger[1,#,*], C. Margreitter[2], C. Tyrchan[1]

[1] Medicinal Chemistry, Research and Early Development, Respiratory and Immunology (R&I), BioPharmaceuticals R&D, AstraZeneca, Gothenburg, Sweden

[2] Computational Chemistry, Discovery Sciences, R&D, AstraZeneca, Gothenburg, Sweden

[¤] Current address: Department of Computer Science, Vrije Universiteit, De Boelelaan 1105, 1081 HV Amsterdam, The Netherlands

[#] Shared first authors

[*] Correspondence: eva.nittinger@astrazeneca.com

A-B-AB SPLITTING STRATEGY

The idea behind this splitting strategy is to show if additive compounds can be predicted more easily based on their matched pair compounds than nonadditive compounds.

Due to the random order in the matched square, any of the four compounds can be considered as ‘AB’. Within the matched square two transformation are available: A and B.

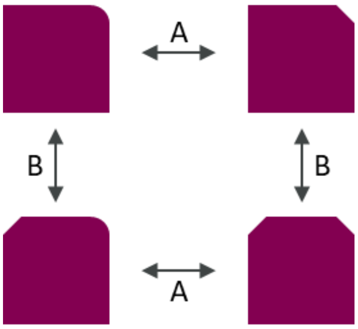

Figure 1. Schematic view of a DTC with two transformations indicated as ‘A’ and ‘B’.

Irrespective of which compound is assigned as ‘AB’, if two other compounds of the cycle are available, the information about both transformations A and B is included. For the nonadditive compounds, there is a clear classification as test compound. Thus, the following strategy is applied to generate the ‘A-B-AB’ nonadditive splitting:

1. Select all compounds with significant NA.
2. Select all DTC in which the NA compounds from 1. appear.
3. Selecting the NA compound from 1. as AB if a DTC from 2. is available where at least two compounds are considered additive, i.e. below the significant threshold. Compounds A and B do not need to be unique, i.e. only appearing in one DTC. Information from up to five DTCs was used for constructing test/training data for NA compounds.

Pseudo-code for selection of nonadditive AB compounds:

```
Get all NA cpds
For each NA cpd:
```

```
    Get all DTC in which it appears
    DTC_count = 0
    For each DTC, while DTC_count < 5:
        Get all 4 cpds and remove the NA cpd
        Check remaining cpds themselves are additive
        If ≥ 2 cpds remain:
            Assign NA cpd to test set
            Assign additive cpds as training
            DTC_count += 1
```

For the additive compounds to be separated into A-B-AB, no clear identification for test is available, since all compounds are additive. Therefore, the following strategy was applied:

1. Select all additive compounds not yet assigned to nonadditive test or training data.
2. Select all DTC in which the compounds from 1. appear.
3. Store compounds from 1. and 2. if a DTC from 2. is available where at least two compounds are considered additive, i.e. below the significant threshold. Compounds A and B do not need to be unique, i.e. only appearing in one DTC.
4. Randomize the list of compounds.
5. Assigning compounds to test data if
    a. The compound is not in the additive training data.
    b. The compound has at least two additive compounds in a DTC which are not yet assigned to either test or training data.
    c. If 20 % of the total number of additive compounds, i.e. training set from the selection of nonadditive A-B-AB and all remaining additive compounds, has not been reached.
6. Assign compounds to training data that are additive and in a DTC selected by 5.
7. All remaining cpds are considered as training if they have not been assigned as test cases.

Pseudo-code for selection of <u>additive</u> AB compounds:

```
Add_cpd_list = []
Add_training_set = []
Add_test_set = []
Get all additive cpds not yet assigned to test or training NA
For each additive cpd:
  Get all DTC in which it appears
  For each DTC:
       Get all 4 cpds and remove the additive cpd
       Check remaining cpds themselves are additive
       If ≥ 2 cpds remain:
            Add_cpd_list append cpds
Randomize Add_cpd_list
For each cpd_X in Add_cpd_list:
  If cpd_X is not in Add_training_set and
  If DTC cpds of cpd_X are and
  If ≥ 2 DTC cpds of cpd_X are additive and
       not in Add_training_set or Add_test_set and
  If Add_test_set < 20 % of all additive compounds:
       Add_test_set append cpd_X
       Add_training_set append DTC cpds of cpd_X
  Else:
       Add_training_set append cpd_X
```

Due to the random selection of compounds (Step 4) to be considered for the additive test set, this randomization is done twice with different random seeds to see any performance difference just based on splitting.

Table S 1. Overview of different models trained for each selected ChEMBL data set

Model ID	Data	Training	Test ID	Test	Rdm seed
**1**	DTC	80 % nonsig	a	20 % nonsig	
			b	all NA cpds	
**2**	DTC	80 % nonsig + Q1 NA cpds	a	mixin NA cpds	
**3**	DTC	80 % nonsig + median NA cpds	a	mixin NA cpds	
**4**	DTC	80 % nonsig + Q3 NA cpds	a	mixin NA cpds	
**5**	all	80 % nonsig	a	20 % nonsig	
			b	all NA cpds	

**6**	all	80 % nonsig + Q1 NA cpds	a	mixin NA cpds	
**7**	all	80 % nonsig + median NA cpds	a	mixin NA cpds	
**8**	all	80 % nonsig + Q3 NA cpds	a	mixin NA cpds	
**9**	DTC	80 % A-B cpds	a	test additive AB cpds	4
			b	NA AB cpds	
			c	remaining NA cpds	
**10**	DTC	80 % A-B cpds	a	test additive AB cpds	7
			b	NA AB cpds	
			c	remaining NA cpds	
**11**	all	80 % A-B cpds + 80 % nonsig	a	test additive AB cpds	4
			b	NA AB cpds	
			c	remaining NA cpds	
			d	20 % nonsig	
**12**	all	80 % A-B cpds + 80 % nonsig	a	test additive AB cpds	7
			b	NA AB cpds	
			c	remaining NA cpds	
			d	20 % nonsig	

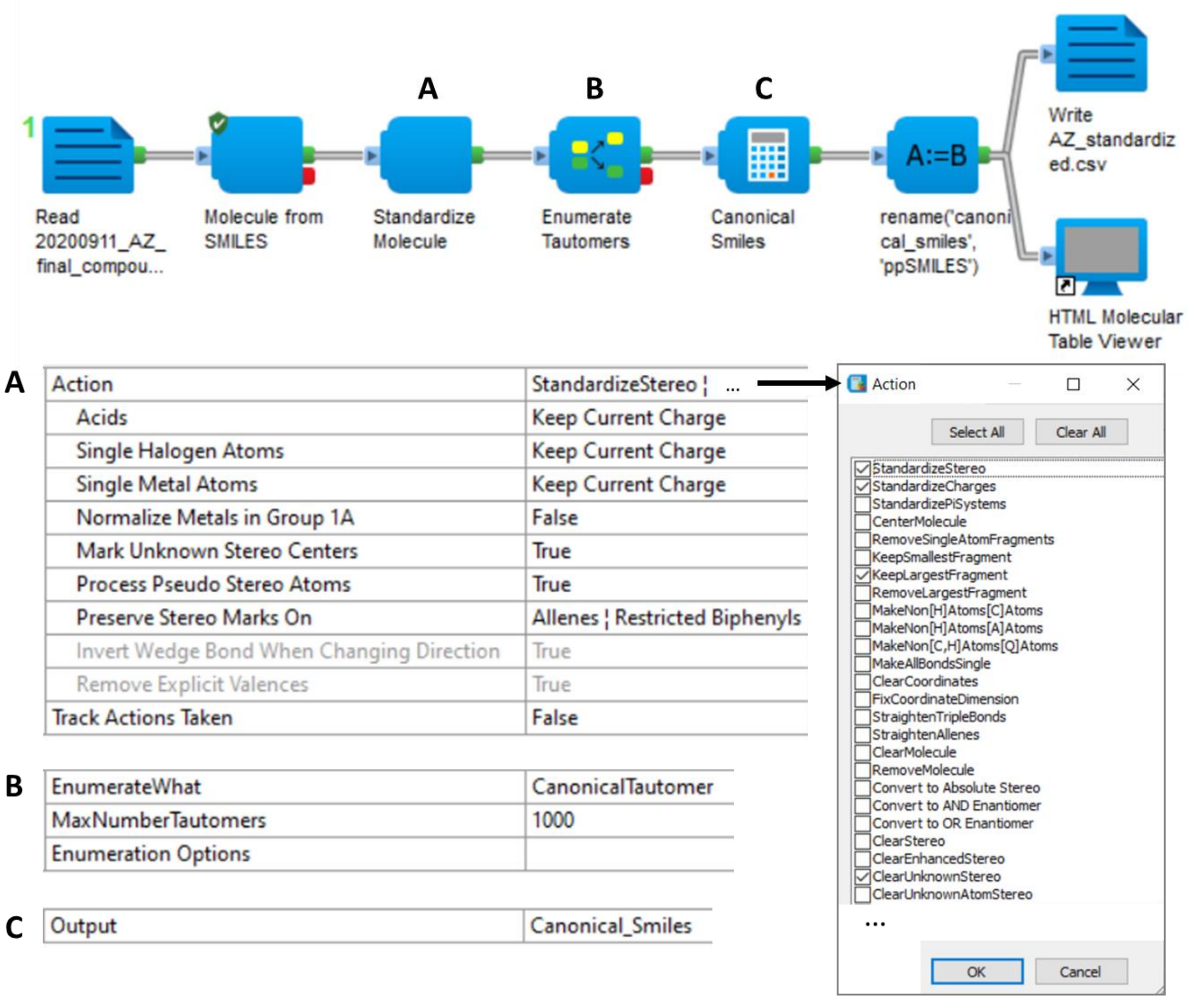

Figure S 1. PipelinePilot standardization protocol used for inhouse and ChEMBL SMILES; further options for components A and B were used as given by default.

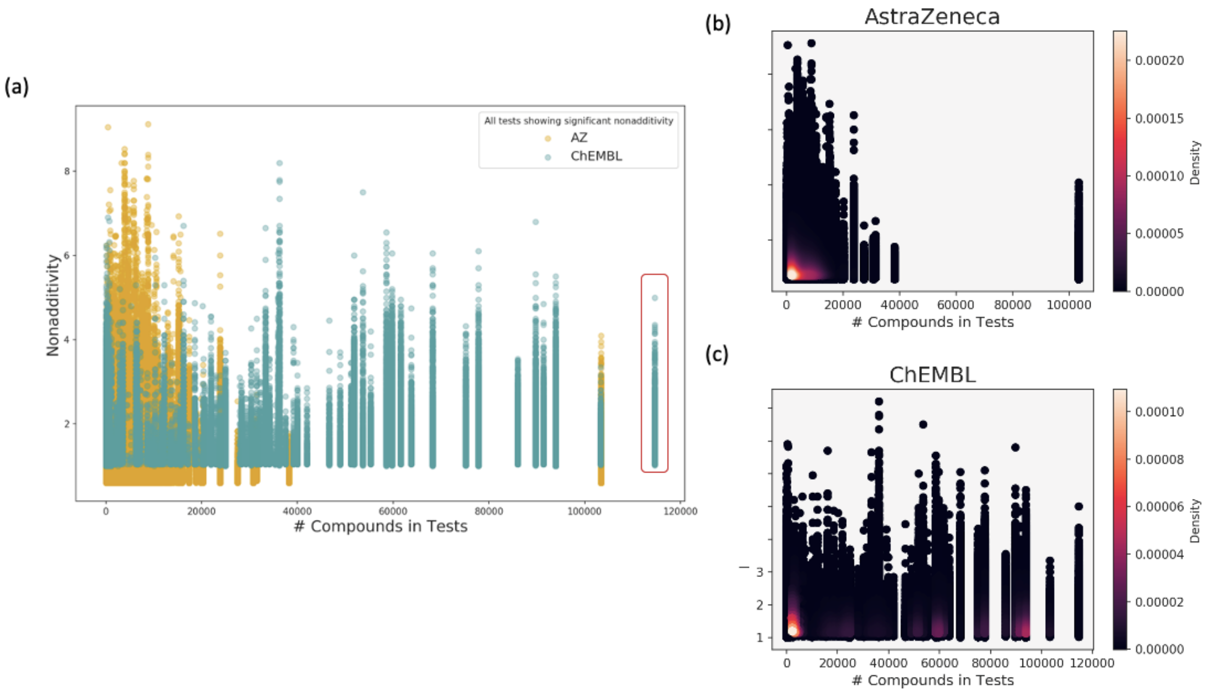

Figure S 2. (a) Distribution of the tests from AZ (yellow) and ChEMBL (blue) based on the size of the test and obtained NA values overlaid. CHEMBL1794483 test is highlighted in red. Density distribution separately for AZ (b) and ChEMBL (c) tests.

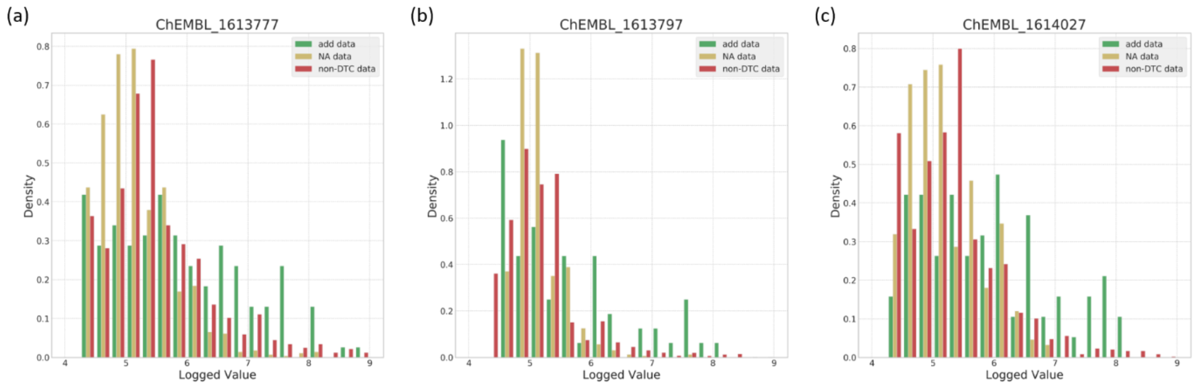

Figure S 3. pIC50 coverage of selected ChEMBL data sets used for QSAR prediction models. Green: additive compounds, yellow: nonadditive compounds, red: non-DTC compounds. Nonadditive compounds have a significant NA value > 1.0 log unit.

Table S 2. Model performance for ChEMBL1614027. Random Forest (RF) and Support Vector Machine (SVM) were trained for models 1-8. A PLS models (model ID 13/14) was trained based on DTC and all data.

**Model ID**	**Data**	**Training data**	**# training**	**Test ID**	**Test data**	**# test**	**algorithm**	**R^2 (RF/SVM)**	**RMSE (RF/SVM)**	**Rdm seed**
**1**	DTC	80 % nonsig	692	a	20 % nonsig	173	RF/SVM	0.598 / 0.602	0.364 / 0.362	
				b	all NA cpds	76	RF/SVM	-0.479 / -0.463	1.256 / 1.249	
**2**	DTC	80 % nonsig + Q1 NA cpds	697	a	mixin NA cpds	58	RF/SVM	-0.605 / -0.59	1.342 / 1.335	
**3**	DTC	80 % nonsig + median NA cpds	701	a	mixin NA cpds	58	RF/SVM	-0.561 / -0.569	1.323 / 1.327	
**4**	DTC	80 % nonsig + Q3 NA cpds	710	a	mixin NA cpds	58	RF/SVM	-0.551 / -0.586	1.319 / 1.334	
**5**	all	80 % nonsig	2240	a	20 % nonsig	560	RF/SVM	0.336 / 0.317	0.567 / 0.574	
				b	all NA cpds	76	RF/SVM	-0.355 / -0.428	1.202 / 1.234	
**6**	all	80 % nonsig + Q1 NA cpds	2255	a	mixin NA cpds	19	RF/SVM	-0.446 / -0.747	1.27 / 1.396	
**7**	all	80 % nonsig + median NA cpds	2269	a	mixin NA cpds	19	RF/SVM	-0.467 / -0.724	1.279 / 1.386	
**8**	all	80 % nonsig + Q3 NA cpds	2297	a	mixin NA cpds	19	RF/SVM	-0.526 / -0.702	1.304 / 1.377	
**9**	DTC	80 % A-B cpds	692	a	test additive AB cpds	173	RF	0.61	0.366	4
				b	NA AB cpds	39	RF	-0.617	1.385	
				c	remaining NA cpds	37	RF	-0.271	1.082	
**10**	DTC	80 % A-B cpds	692	a	test additive AB cpds	173	RF	**0.69**	**0.315**	7
				b	NA AB cpds	39	RF	*-0.66*	*1.404*	
				c	remaining NA cpds	37	RF	-0.219	1.059	
**11**	all	80 % A-B cpds + 80 % nonsig	2240	a	test additive AB cpds	173	RF	0.589	0.379	4
				b	NA AB cpds	39	RF	-0.514	1.34	
				c	remaining NA cpds	37	RF	-0.113	1.012	
				d	20 % nonsig	387	RF	0.219	0.677	
**12**	all	80 % A-B cpds + 80 % nonsig	2240	a	test additive AB cpds	173	RF	**0.63**	**0.344**	7
				b	NA AB cpds	39	RF	-0.578	1.368	
				c	remaining NA cpds	37	RF	-0.065	0.99	
				d	20 % nonsig	387	RF	0.198	0.686	
**13**	DTC	80 % nonsig	692	a	20 % nonsig	173	PLS	0.537	0.39	
				b	all NA cpds	76	PLS	-0.6	1.306	
**14**	all	80% nonsig	2240	a	20 % nonsig	560	PLS	0.246	0.603	
				b	all NA cpds	76	PLS	-0.394	1.219	

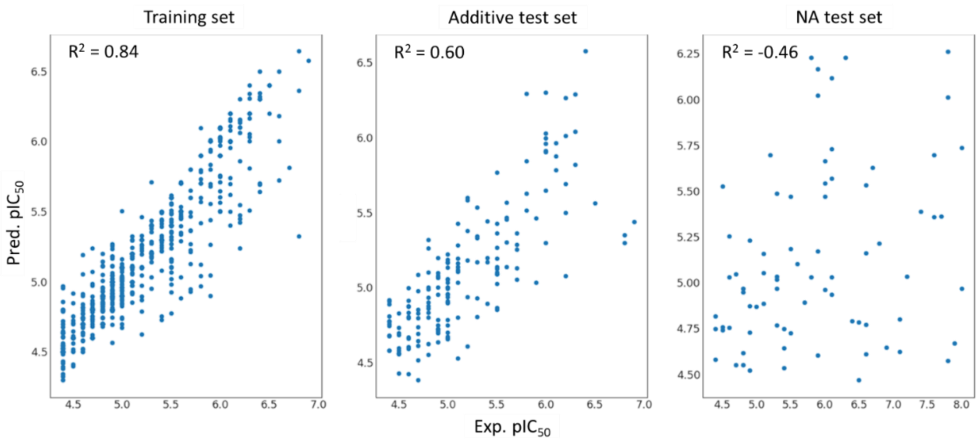

Figure S 4. SVM correlation plots for ChEMBL1614027.

Table S 3. Random Forest model performance for ChEMBL1613777.

Model ID	Data	Training data	# training	Test ID	Test data	# test	R^2	RMSE	Rdm seed
**1**	DTC	80 % nonsig	886	a	20 % nonsig	222	**0.564**	**0.442**	
				b	all NA cpds	153	-0.431	1.296	
**2**	DTC	80 % nonsig + Q1 NA cpds	893	a	mixin NA cpds	126	-0.443	1.29	
**3**	DTC	80 % nonsig + median NA cpds	900	a	mixin NA cpds	126	-0.443	1.29	
**4**	DTC	80 % nonsig + Q3 NA cpds	913	a	mixin NA cpds	126	-0.384	1.264	
**5**	all	80 % nonsig	2675	a	20 % nonsig	669	0.22	0.684	
				b	all NA cpds	153	-0.339	1.254	
**6**	all	80 % nonsig + Q1 NA cpds	2694	a	mixin NA cpds	80	-0.234	1.203	
**7**	all	80 % nonsig + median NA cpds	2712	a	mixin NA cpds	80	-0.218	1.195	
**8**	all	80 % nonsig + Q3 NA cpds	2748	a	mixin NA cpds	80	-0.168	1.17	
**9**	DTC	80 % A-B cpds	918	a	test additive AB cpds	190	0.535	0.387	4
				b	NA AB cpds	127	-0.388	1.265	
				c	remaining NA cpds	26	-0.423	1.318	
**10**	DTC	80 % A-B cpds	920	a	test additive AB cpds	188	0.455	0.413	7
				b	NA AB cpds	127	-0.394	1.268	
				c	remaining NA cpds	26	-0.405	1.31	
**11**	all	80 % A-B cpds + 80 % nonsig	2706	a	test additive AB cpds	190	0.433	0.428	4
				b	NA AB cpds	127	-0.383	1.263	
				c	remaining NA cpds	26	-0.236	1.229	
				d	20 % nonsig	448	0.11	0.806	
**12**	all	80 % A-B cpds + 80 % nonsig	2708	a	test additive AB cpds	188	**0.439**	**0.419**	7
				b	NA AB cpds	127	-0.329	1.238	
				c	remaining NA cpds	26	-0.261	1.241	
				d	20 % nonsig	448	0.129	0.797	

Table S 4 Random forest model performance for ChEMBL1613797.

**Model ID**	**Data**	**Training data**	**# training**	**Test ID**	**Test data**	**# test**	**R^2**	**RMSE**	**Rdm seed**
**1**	DTC	80 % nonsig	509	a	20 % nonsig	128	0.047	0.407	
				b	all NA cpds	64	-0.286	1.142	
**2**	DTC	80 % nonsig + Q1 NA cpds	513	a	mixin NA cpds	51	-0.237	1.179	
**3**	DTC	80 % nonsig + median NA cpds	516	a	mixin NA cpds	51	-0.226	1.174	
**4**	DTC	80 % nonsig + Q3 NA cpds	522	a	mixin NA cpds	51	-0.25	1.185	
**5**	all	80 % nonsig	4924	a	20 % nonsig	1231	0.05	0.578	
				b	all NA cpds	64	-0.212	1.109	
**6**	all	80 % nonsig + Q1 NA cpds	4940	a	mixin NA cpds	3	-0.233	0.499	
**7**	all	80 % nonsig + median NA cpds	4955	a	mixin NA cpds	3	-0.429	0.538	
**8**	all	80 % nonsig + Q3 NA cpds	4985	a	mixin NA cpds	3	-0.143	0.481	
**9**	DTC	80 % A-B cpds	515	a	test additive AB cpds	122	0.025	0.385	4
				b	NA AB cpds	28	-0.554	1.259	
				c	remaining NA cpds	36	-0.123	0.983	
**10**	DTC	80 % A-B cpds	510	a	test additive AB cpds	122	0.102	0.331	7
				b	NA AB cpds	28	-0.6	1.277	
				c	remaining NA cpds	36	-0.103	0.974	
**11**	all	80 % A-B cpds + 80 % nonsig	4929	a	test additive AB cpds	122	0.035	0.383	4
				b	NA AB cpds	28	-0.607	1.28	
				c	remaining NA cpds	36	-0.117	0.981	
				d	20 % nonsig	1104	0.048	0.595	
**12**	all	80 % A-B cpds + 80 % nonsig	4924	a	test additive AB cpds	122	0.039	0.342	7
				b	NA AB cpds	28	-0.574	1.267	
				c	remaining NA cpds	36	-0.119	0.981	
				d	20 % nonsig	1104	0.046	0.595	

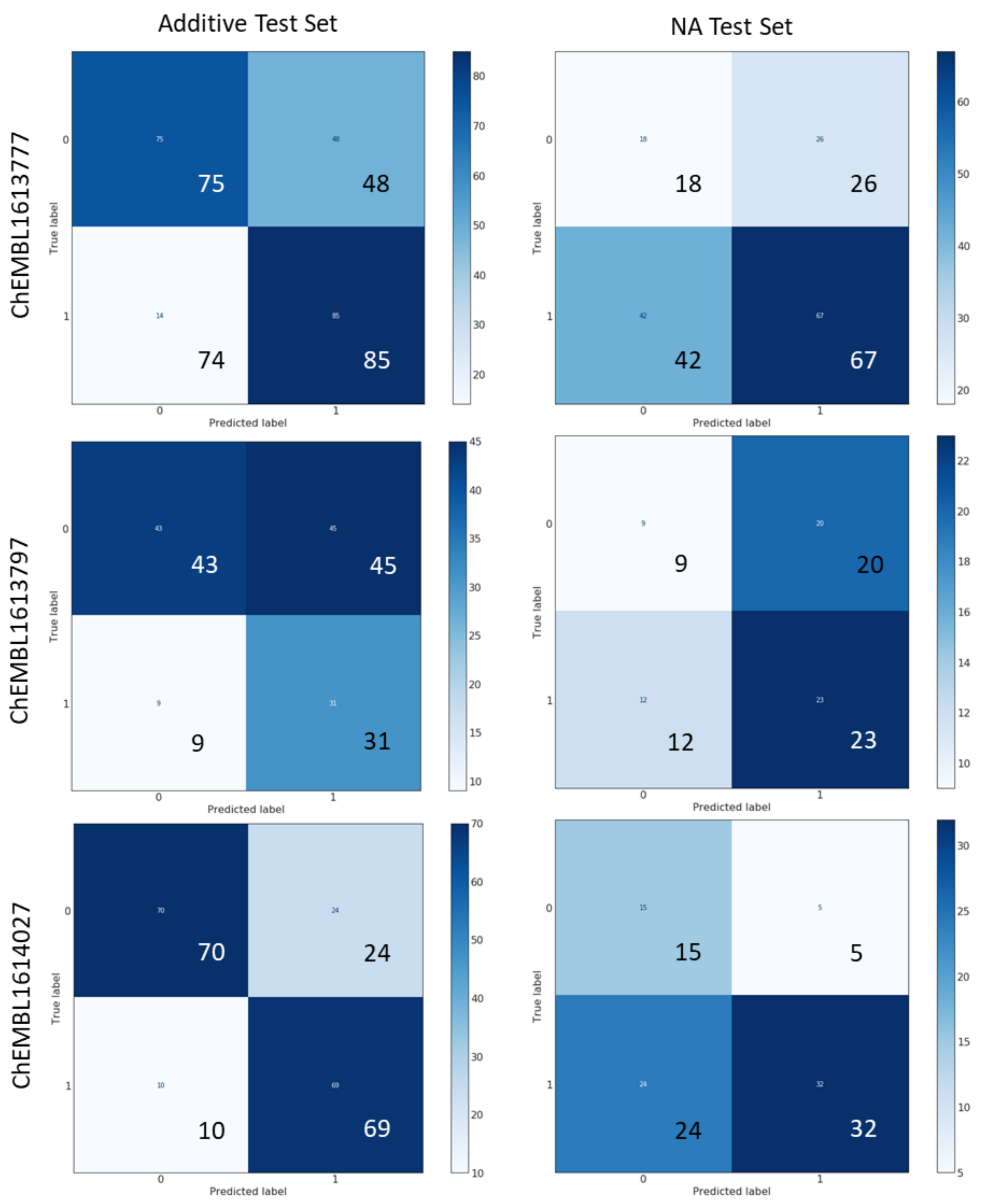

Figure S 5. Confusion matrices for the binary classification of additive and nonadditive test sets. Predictions were done using RF models, binary classification was based on pIC_{50} = 5.

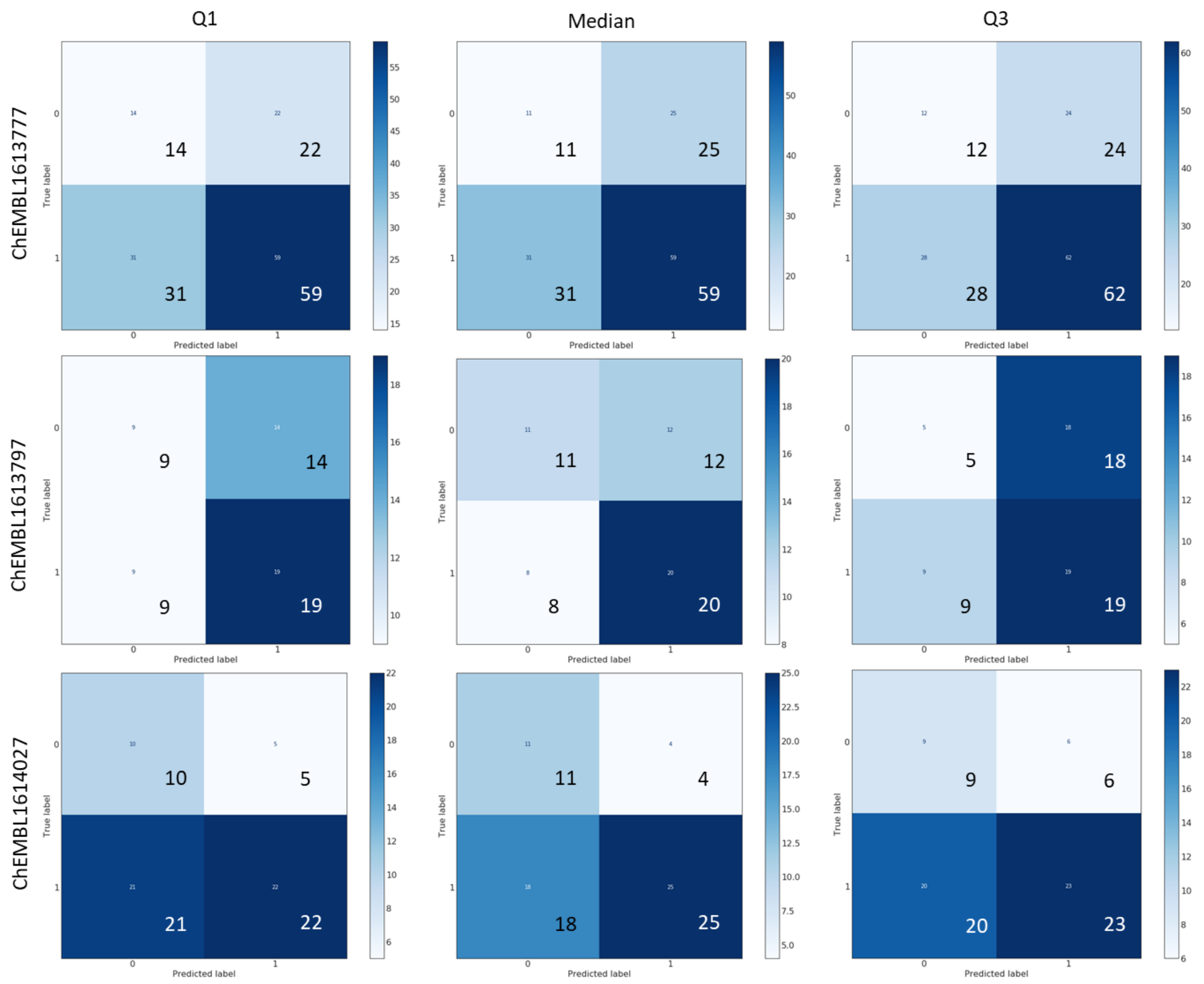

Figure S 6. Confusion matrices for binary classification for the ‘mixin’ data sets. Predictions were done using RF models, binary classification was based on pIC_{50} = 5.
